# An imbalance-aware deep neural network for early prediction of preeclampsia

**DOI:** 10.1371/journal.pone.0266042

**Published:** 2022-04-06

**Authors:** Rachel Bennett, Zuber D. Mulla, Pavan Parikh, Alisse Hauspurg, Talayeh Razzaghi

**Affiliations:** 1 School of Industrial and Systems Engineering, University of Oklahoma, Norman, Oklahoma, United States of America; 2 Department of Obstetrics and Gynecology, and Office of Faculty Development, Paul L. Foster School of Medicine, Texas Tech University Health Sciences Center El Paso, El Paso, Texas, United States of America; 3 Department of Public Health, Texas Tech University Health Sciences Center, Lubbock, Texas, United States of America; 4 Division of Maternal Fetal Medicine, University of Oklahoma Health Science Center, Oklahoma City, Oklahoma, United States of America; 5 Division of Maternal-Fetal Medicine, Department of Obstetrics, Gynecology, and Reproductive Sciences, University of Pittsburgh School of Medicine, Pittsburgh, Pennsylvania, United States of America; University of Bonab, IRAN, ISLAMIC REPUBLIC OF

## Abstract

Preeclampsia (PE) is a hypertensive complication affecting 8-10% of US pregnancies annually. While there is no cure for PE, aspirin may reduce complications for those at high risk for PE. Furthermore, PE disproportionately affects racial minorities, with a higher burden of morbidity and mortality. Previous studies have shown early prediction of PE would allow for prevention. We approached the prediction of PE using a new method based on a cost-sensitive deep neural network (CSDNN) by considering the severe imbalance and sparse nature of the data, as well as racial disparities. We validated our model using large extant rich data sources that represent a diverse cohort of minority populations in the US. These include Texas Public Use Data Files (PUDF), Oklahoma PUDF, and the Magee Obstetric Medical and Infant (MOMI) databases. We identified the most influential clinical and demographic features (predictor variables) relevant to PE for both general populations and smaller racial groups. We also investigated the effectiveness of multiple network architectures using three hyperparameter optimization algorithms: Bayesian optimization, Hyperband, and random search. Our proposed models equipped with focal loss function yield superior and reliable prediction performance compared with the state-of-the-art techniques with an average area under the curve (AUC) of 66.3% and 63.5% for the Texas and Oklahoma PUDF respectively, while the CSDNN model with weighted cross-entropy loss function outperforms with an AUC of 76.5% for the MOMI data. Furthermore, our CSDNN model equipped with focal loss function leads to an AUC of 66.7% for Texas African American and 57.1% for Native American. The best results are obtained with 62.3% AUC with CSDNN with weighted cross-entropy loss function for Oklahoma African American, 58% AUC with DNN and balanced batch for Oklahoma Native American, and 72.4% AUC using either CSDNN with weighted cross-entropy loss function or CSDNN with focal loss with balanced batch method for MOMI African American dataset. Our results provide the first evidence of the predictive power of clinical databases for PE prediction among minority populations.

## Introduction

Preeclampsia (PE) spectrum disorders occur in pregnant women and are generally defined by new onset hypertension and proteinuria after week 20 of gestation. PE afflicts 8–10% of the approximately 4 million yearly pregnancies in the US [1]. Of those women who survive, PE is associated with long-term health effects, such as increased risk of heart disease, stroke, and diabetes [2]. Children of women with PE also have increased risk of long-term cardiovascular illness [3]. Studies have shown that the low-dose aspirin early in pregnancy can reduce the occurrence of PE in pregnant women who are at high risk [4]. Stratification of women at the highest risk of PE would allow clinicians to provide primary prevention at the right time.

Several statistical and machine learning (ML) models have been developed. A comprehensive overview of previous studies is given in Table 1. Kenny et al. (2014) [5] and Sandstrom et al. (2019) [6] applied logistic regression to predict PE among nulliparous women. Moreira et al. (2017) [7] and Sufriyana et al. (2020) [8] have successfully used random forest classifiers to predict PE; however, the random forest algorithm and its variable importance measures tend to show bias in the presence of predictor variables with many categories and variables with different scale of measurement [9]. Marić et al. (2020) [10] have proposed the use of the elastic net model for PE prediction, but their study focused on a single high-risk referral hospital which included a higher occurrence of PE than in the general population.

**Table 1:** Summary of early-onset PE prediction previous studies with methods they used. MImp.: Missing imputation technique, R: Removal technique, EM: Expected maximization, M: Mean Imputation, FFS: Forward Feature Selection, BFS: Backward Feature Selection, CBF: Correlation Based Feature Selection, PCA: Principal Componant Analysis, Imb.: class imbalance method, OS: oversampling, WT: class-weight adjustment, ST: SMOTE, LR: Logistic regression, EN: Elastic Net, DT: Decision Tree, RF: Random Forest, SVM: Support vector machine, ANN: Artificial Neural Network, GB: Gradient Boosting, EL: Ensemble Learning, NB: Naïve Bayes, KNN: K Nearest Neighbor, KS: Kappa statistics, SN: Sensitivity, SP: Specificity, ACC: Accuracy, PR: Precision, GM: G-mean, FM: F-measure, BR: Brier Score.

Authors (Year)	MImp.	Feature Selection	Imb.	ML Method	Performance Measure
Wang et al. (2022) [46]	-	PCA	-	SVM, CNN, NB	ACC, PR, SN, FM
Wanriko et al. (2021) [53]	-	-	ST	LR, DT, RF, SVM, ANN, NB	ACC
Li et al. (2021) [48]	-	XGBoost	WT	LR, GB, RF, SVM	ACC, AUC, PR, SN, FM, BR
Manoochehri et al. (2021) [49]	-	-	-	LR, DT, RF, SVM, KNN	ACC, SN, SP
Guo et al. (2021) [42]	-	CBF	-	DT, ANN, Adaboost	ACC, AUC, PR, SN, SP
Maric et al. (2020) [10]	M	FFS	-	EN, LR, GB	AUC, SN
Sufriyana et al. (2020) [8]	R	FFS	OS	LR, DT, RF, SVM, ANN, EL	AUC, PR, SN, SP
Sandstrom et al. (2019) [6]	R	BFS	-	LR	AUC, SN
Moreira et al. (2017) [7]	R	-	-	DT, RF, NB	AUC, PR, SN, FM, KS
Kenny et al. (2014) [5]	R	BFS	-	LR	AUC, PR, SN
Caradeux et al (2013) [45]	-	BFS	-	LR	AUC, PR, SN
Parra-Cordero et al. (2013) [44]	-	-	-	LR	AUC, SN
Scazzocchio et al. (2013) [54]	R	FFS	-	LR	AUC
North et al. (2011) [55]	EM	BFS	-	LR	AUC
Odibo et al. (2011) [56]	-	-	-	LR	AUC, PR, SN
Yu et al. (2005) [57]	-	BFS	-	LR	AUC, SN, SP

Previous studies have shown that the mothers’ race and ethnicity are significant risk factors for PE [11]. Minority women experience severe morbidity and mortality rates as high as four times that of their Caucasian counterparts during pregnancy and postpartum [12–16], with several studies reporting an increased incidence of PE among African American (AA) and American Indian/Native American (NA) women [17–23]. AA women have a higher risk of a prolonged length of stay in the hospital and progression to severe forms of PE [24]. Long-term follow-up of patients with PE indicates a high recurrence risk in future pregnancies and a two to eight-fold risk of cardiovascular disease [25, 26], affecting minorities more frequently and with more adverse maternal and neonatal outcomes [27]. Delivery may reduce the risk for adverse outcomes for the mother, but premature delivery presents many complications for the baby, having consequences for the whole family. Unfortunately, minority women often initiate prenatal care later [28], limiting the lead time for physicians to assess each patient’s individual risk for PE, negatively affecting care delivery effectiveness [13].

Existing works have rarely addressed the inherent sparsity and large number of categorical variables in the data available in large clinical databases, the presence of noisy and missing data, and the skewed distribution of observations (known as imbalanced data) among preeclamptic and healthy individuals. Therefore, these ML models may not be reliable, or interpretable (due to the sparsity and presence of a large number of categorical variables). The severity of these issues is even higher for AA and NA populations as confirmed by our study on a number of PE datasets. Modern ML models, have been extremely effective in dealing with these issues in application of predictive modeling [29, 30]. In particular imbalance-aware models can handle imbalanced data during the learning process [31–35].

To our knowledge, there are no reliable ML-based decision support approaches specific to PE prediction, particularly for AA and NA populations. There is no study that has investigated the risk factors of PE and developed predictive models for AA and NA populations using advanced ML techniques, particularly Deep neural networks (DNN) [36, 37]. DNNs have been useful for large, high-dimensional datasets [38–40]. They are flexible to being extended for imbalanced classification problems [41].

In the absence of heterogeneous and multimodal data such as placental mRNA Samples [42], Uterine artery Doppler measurements [5, 43–45], medications [10], and images [46] to predict PE, the contributions of this paper are multi-fold as stated below.

1. First, using the chi-square feature selection method, we identify the significant clinical and demographic attributes associated with developing PE among women from AA and NA populations as well as general populations using large datasets. There is no existing work that has studied the PE risk prediction using ML for minority populations.
2. Second, we construct a new cost-sensitive deep neural network (CSDNN) prediction model capable of identifying women with high suspicion of developing PE and estimating their associated risk (probability) using highly imbalanced and high-dimensional sparse datasets. In particular, we extend the idea of using focal loss to classify sparse imbalanced PE datasets, where focal loss has been primarily utilized for object detection problems [47] in the literature. To the best of the authors knowledge, there is no advanced deep neural network algorithm that takes into consideration the imbalanced and sparse nature of the preeclampsia prediction problem.
3. Lastly, we demonstrate the effectiveness and impact of the proposed scheme through a rich array of datasets which represents a diverse cohort of both AA and NA populations such as Texas Public Use Data Files (PUDF), Oklahoma PUDF, and the Magee Obstetric Medical and Infant (MOMI) databases. Our work is distinguished from previous works that studied small EHR datasets (with few hundreds or less patients in their cohort [7, 43, 48, 49]). The Texas data includes a total of 360,943 patients delivered at the hospital. Of those, 14,375 (3.98%) developed PE. The Oklahoma data contains a total of 84,632 women who delivered in-hospital, of which 4,721 (5.58%) developed PE. The MOMI data includes in total 31,431 women who delivered in-hospital, of which 2,743 (8.73%) developed PE.
4. Furthermore, in order to improve the accuracy of our proposed models using MOMI data, we have added a new variable for each patient which represents the number of incidents of spikes in blood pressure within the first 14 weeks. However, we were not able to use this variable with Texas and Oklahoma datasets, but using the MOMI dataset our work intends to identify the PE patients as early as possible with higher accuracy by considering the incidents of spikes in the blood pressure within the first 14 weeks compared to the existing studies.

## Methods

### Artificial neural networks

Artificial Neural Networks (ANN) originated in the 1940s, with the McCulloch-Pitts Neuron [50]. The idea of “artificial neurons” is inspired by the human brain, in which a neuron takes “input” in the form of signals from surrounding cells, and will only activate in the form of an electrical spike if the combined signals passes a threshold level. An artificial neuron mimics this behavior by taking a series of features *x*, multiplying each by an individually chosen weight *w*, and then adds the result to a bias term *b* before summing them together to calculate if a pre-defined threshold is met, which allows for classification.

Later versions of ANN adapted the artificial neuron to represent more complicated functions by linking them together into a multilayer perceptron (MLP) or feedforward ANN [51]. The MLP is typically composed of multiple layers, each layer containing a pre-defined number of neurons, or nodes. These layers can be subdivided into three separate types: the input layer, which takes each feature *x* as input; a number of hidden layers (the number of layers here denotes the depth of the network), which performs the previously seen linear computation on each input before passing the output to the next layer; and, the output layer, which returns the final prediction. Each node in a layer is connected to every node in the next layer, making a fully-connected neural network where the final prediction is a functional composition of each layer.

These functions each take the form of:

$$\begin{array}{c} f(x,w,b)=w*x+b \end{array}$$ (1)

Where *x* are the input features, *w* are the weights of each node in the layer, and *b* are accompanying bias term. More modern versions of neural networks add non-linearity through the use of activation functions [52]. The most commonly-used activation function is the sigmoid activation given by

$$\begin{array}{c} sigmoid\;:\;a\left(z\right)=\frac{1}{1+e^{-z}} \end{array}$$ (2)

Where *z* represents the linear output of the node. The downside of this activation function is that it can saturate, meaning that if the output is too large or small the gradient can become close to 0 which negatively affects the ability of the network to update the parameters. To overcome this issue, the sigmoid function has been improved through using the related hyperbolic tangent function given by

$$\begin{array}{c} tanh\;:\;a\left(z\right)=\frac{2}{1+e^{-2z}}-1 \end{array}$$ (3)

This function outputs values between -1 and 1 and has a significantly steeper gradient which makes it easier for training than using the sigmoid function. Another common activation function is the rectified linear unit (ReLU) function:

$$\begin{array}{c} ReLU\;:\;a(z)=max(0,z) \end{array}$$ (4)

*ReLU* tends to converge faster than *sigmoid* or *tanh* [58] which makes the learning of a neural network more efficient. Due to the ability to learn complex non-linear functions, neural networks have been used successfully in a variety of machine learning problems, such as image recognition [59, 60], machine translation [61, 62], speech recognition [63, 64], weather forecasting [65], credit scoring [66], and cancer detection [67–69].

### Back-propagation and gradient-based learning

The ANN models employ specific optimization processes to obtain the parameters (w*, b*) at each layer, which is called the back-propagation stage [70]. Stochastic gradient descent (SGD) [71], Adaptive Moment Estimation (Adam) [72], NAdam [73], and root mean squared propagation (RMSProp) [74] are the most common optimizers. We use the RMSProp, Adam, and NAdam methods in the back-propagation for each dataset due to their superior performance in computational efficiency [73–75]. We select the best optimizer for each dataset using model selection algorithms explained below.

The parameters (w*, b*) is calculated using these optimization algorithms at each epoch. The number of epochs used in the model is another hyperparameter. Too few epochs may lead to the model not learning the data and too many may result in over-fitting, limiting generalization to other datasets [36]. The best number of epochs is identified through the early stopping method, in which the risk of overtraining is reduced by stopping training after the validation error is stabilized or no further improvement is made.

### Cost-sensitive neural networks

Despite the success of neural networks in a variety of applications, their use might be challenging due to the distribution of the given dataset. In classification, many machine learning algorithms, including neural networks, assume that the distribution of classes is roughly the same. When this assumption is violated, the neural network can best reduce the misclassification cost by outputting the majority class in every case. This results in a model with high accuracy, but with no ability to distinguish between classes [76].

Our method removes this assumption using a cost-sensitive learning approach. In this approach, originally proposed by Kukar [77], the cost function is modified such that different costs are associated with the true value of any given sample. Two specific loss functions were employed: weighted cross-entropy and focal loss functions.

### Weighted cross-entropy loss

In neural networks, the cross-entropy (*CE*) loss function is usually used for binary classification problems which is defined by

$$\begin{array}{c} CE\left(p,y\right)=\{\begin{array}{cc} -\text{log}\;\left(p\right) & \text{if}\;y=1\\ -\text{log}\;\left(1-p\right) & \text{otherwise.} \end{array} \end{array}$$ (5)

Where *y* ∈ {±1} is the ground-truth class and *p* ∈ [0, 1] is the model’s estimated probability of the class with label y = 1. This basic loss function can be modified by multiplying the cost of each individual sample by a class specific weight [47], which results in what is referred to as the weighted *CE* (*WCE*) or balanced *CE*, defined by:

$$\begin{array}{c} WCE\left(p,y\right)=\{\begin{array}{cc} -C^{+}\;\text{log}\;\left(p\right) & \text{if}\;y=1\\ -C^{-}\;\text{log}\;\left(1-p\right) & \text{otherwise.} \end{array} \end{array}$$ (6)

Where $C^{+}=\frac{N}{2N^{+}}$, $C^{-}=\frac{N}{2N^{-}}$, and *N*^+^ and *N*^−^ are the sizes of the positive and negative classes respectively. The parameters *C*^+^ and *C*^−^ represents misclassification penalties of samples in the minority (positive) and majority (negative) class respectively. Accordingly, the error cost function is formulated with Eq 7.

$$\begin{array}{c} J\left(w,b\right)=-\frac{1}{N}\left(C^{+}\sum_{\left\{i|y_{i}=1\right\}}^{N^{+}}y_{i}\;\text{log}\;\left(p_{i}\right)+C^{-}\sum_{\left\{j|y_{j}=-1\right\}}^{N^{-}}\left(1-y_{j}\right)\;\text{log}\;\left(1-p_{j}\right)\right) \end{array}$$ (7)

### Focal loss

Focal Loss (*FL*) is an extension of *CE* loss for binary imbalanced classification proposed by Lin et al. [47], and was initially developed for object detection application. The main idea behind the *FL* is to focus training on hard samples while reducing the loss contribution from well-classified and easy samples through adding a modulating factor to the sigmoid CE loss.

Suppose the predicted output from the model for both classes are $\hat{y}={\left[{\hat{y}}_{1},{\hat{y}}_{2}\right]}^{T}$. The sigmoid function calculates the probability distribution for minority and majority classes as *p*_*t*_ = sigmoid(${\hat{y}}_{t}$) = 1/(1 + exp($-{\hat{y}}_{t}$)) where *p*_*t*_ is provided in Eq 8,

$$\begin{array}{c} p_{t}=\{\begin{array}{cc} p & \text{if}\;y=1\\ 1-p & \text{otherwise.} \end{array} \end{array}$$ (8)

The focal loss can be formulated with Eq 9:

$$\begin{array}{c} FL\left(p,y\right)=\{\begin{array}{cc} -{\left(1-p\right)}^{\gamma }\;\text{log}\;\left(p\right) & \text{if}\;y=1\\ -p^{\gamma }\;\text{log}\;\left(1-p\right) & \text{otherwise.} \end{array} \end{array}$$ (9)

where *y* ∈ {±1} is the ground-truth class and *p* ∈ [0, 1] is the model’s estimated probability for the class with label *y* = 1. The parameter *γ* ≥ 0 should be tuned. The modulating factor (1−*p*_*t*_)^*γ*^ is added which reduces the loss contribution from easy examples—in essence, the more confident a model is in its prediction, the less the sample will contribute to the loss. *FL* is equivalent to *CE*, when *γ* = 0. The effect of the modulating factor increases as the *γ* parameter increases [47].

In addition, an *α*-balanced variant of the original focal loss has been developed to further focus on the effective number of samples. The parameter *α*_*t*_ ∈ [0, 1] is defined with Eq 10.

$$\begin{array}{c} \alpha_{t}=\{\begin{array}{cc} \alpha & \text{if}\;y=1\\ 1-\alpha & \text{otherwise.} \end{array} \end{array}$$ (10)

The *α*-balanced variant of the focal loss has shown better performance over the non-*α* balanced form [47]. It is calculated with Eq 11.

$$\begin{array}{c} FL\left(p,y\right)=\{\begin{array}{cc} -\alpha {\left(1-p\right)}^{\gamma }\;\text{log}\;\left(p\right) & \text{if}\;y=1\\ -\left(1-\alpha \right)p^{\gamma }\;\text{log}\;\left(1-p\right) & \text{otherwise.} \end{array} \end{array}$$ (11)

Therefore, the error cost function is provided in Eq 12.

$$\begin{array}{c} J\left(w,b\right)=-\frac{1}{N}\left(\sum_{\left\{i|y_{i}=1\right\}}^{N^{+}}\alpha {\left(1-p_{i}\right)}^{\gamma }\;\text{log}\;\left(p_{i}\right)+\sum_{\left\{j|y_{j}=-1\right\}}^{N^{-}}\left(1-\alpha \right)p_{j}^{\gamma }\;\text{log}\;\left(1-p_{j}\right)\right) \end{array}$$ (12)

We used *WCE* and *α*-balanced focal loss functions to treat imbalanced classification and we represented the deep neural network based on these two loss function using CSDNN-WCE and CSDNN-FL, respectively. We also used the standard deep neural network with CE loss function for comparison purposes, and for simplicity we denote it as DNN throughout this paper.

### Balanced batch training for imbalanced data

For comparison, we also used the balanced batch generator from the scikit-learn Imbalanced-learn library [78]. It utilizes a chosen sampling strategy to balance a dataset prior to generating a batch of data for training. We applied random oversampling with replacement, which randomly selects data samples from the PE class (minority class) and includes them in the training data.

### Chi-square feature selection

Chi-square (*χ*^2^) feature selection [79] selects significant features using the test of independence between the feature and the classes:

$$\begin{array}{c} \chi^{2}=\sum_{i=1}^{m}\sum_{j=1}^{l}\frac{{\left(N_{ij}-E_{ij}\right)}^{2}}{E_{ij}} \end{array}$$ (13)

Where *N*_*ij*_ is the number of samples that belong to class *C*_*i*_ in the *j*^*th*^ interval. *E*_*ij*_ is the expected frequency of *N*_*ij*_ and *l* is the number of intervals. The features with the highest *χ*^2^ values are selected for the predictive model. This method is useful for categorical data, which was ideal for our datasets.

### Performance measures

**Fig 1:**
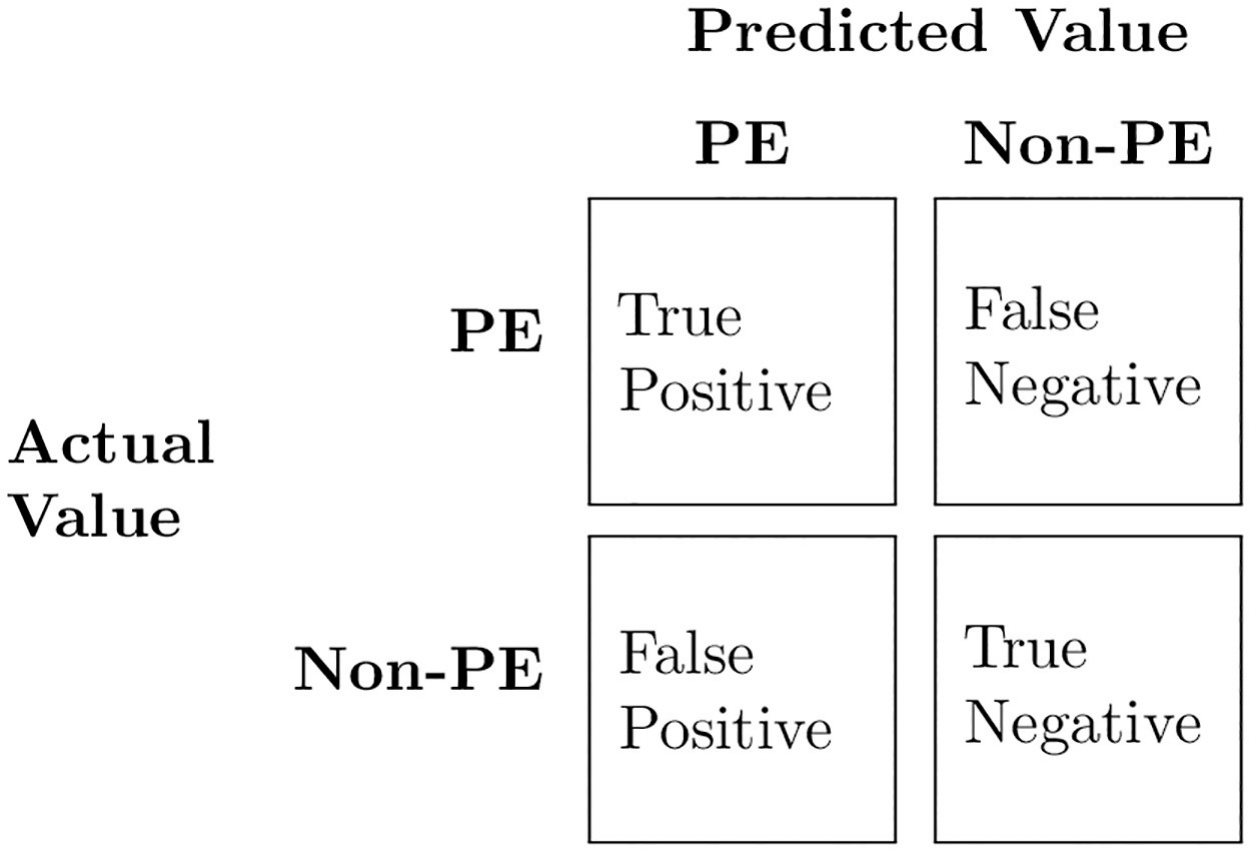
Confusion matrix for binary classification problems.

The most commonly-used performance measures in binary classification tasks are calculated from the confusion matrix (Fig 1).

The number of true positives (TP) represents the number of preeclamptic (PE) patients correctly classified, while true Negatives (TN) is the number of non-preeclamptic (Non-PE) patients classified as Non-PE. The number of false positives (FP) refers to the Non-PE patients classified as PE, while false negatives (FN) represents PE patients classified as Non-PE.

The most common metric in classification tasks is accuracy, provided by Eq 14, which measures the total proportion of correctly classified samples.

$$\begin{array}{c} Accuracy=\frac{TP+TN}{TP+TN+FP+FN} \end{array}$$ (14)

Accuracy is very sensitive to the size of the majority class (Non-PE), and is likely to obtain a misleadingly high accuracy dominated by the majority class pattern while the minority class samples are most likely misclassified. Accuracy does not account for this imbalance, and so we used additional metrics to better understand model performance.

We report Precision, recall, G-mean, and AUC. Precision measures the number of positive values that are actually positive, while recall (or sensitivity) measures what percentage of the positive cases were captured by the model. Specificity refers to the percentage of the negative examples that are truly negative. We also report G-mean, which takes into account both the specificity and sensitivity, as well as the area under the receiver operating characteristic curve (AUC), which measures the balance between the correctly classified positive samples (TP) and incorrectly classified negative samples (FP). The performance metrics were calculated with Eqs 15–18.

$$\begin{array}{c} Precision=\frac{TP}{TP+FP} \end{array}$$ (15)

$$\begin{array}{c} Recall/Sensitivity=\frac{TP}{TP+FN} \end{array}$$ (16)

$$\begin{array}{c} Specificity=\frac{TN}{TN+FP} \end{array}$$ (17)

$$\begin{array}{c} G-mean=\sqrt{Sensitivity*Specificity} \end{array}$$ (18)

### Data preparation

We compare and validate the ML models using three datasets including the 2013 Texas Inpatient Public Use Data File (PUDF) (case 1), the 2017–2018 Oklahoma PUDF sets (case 2), and a granular research hospital data which is the Magee Obstetric Medical and Infant (MOMI) data (case 3). These state and national datasets that represent a diverse cohort of African American and Native American populations, allowing us to examine racial disparities in PE outcome specifically in our analysis. The PUDFs exclude information that could identify patients directly or indirectly. Access to these data files is given to users after submission and approval of the Data Use Agreement. The University of Oklahoma institutional ethical review board approval was obtained for this study (#12718, 07/20/2020). The characteristics of these datasets are explained in this section.

#### Case 1: Texas PUDF

The 2013 Texas Inpatient PUDF has patient-level information related to inpatient hospital stays collected from all state-licensed hospitals except those that are exempt from reporting.“Exempt” hospitals include those located in a county with a population less than 35,000, or those located in a county with a population more than 35,000 and with fewer than 100 licensed hospital beds and not located in an area that is delineated as an urbanized area by the United States Bureau of the Census [80]. This data is maintained and extracted from the Texas Department of State Health Services’ Hospital Discharge Database [81].

The Texas PUDF includes both sociodemographic and clinical information about each patient. The clinical information in particular is contained within up to 25 admission and discharge diagnosis codes for each patient, and are defined by the The International Classification of Diseases, Ninth Revision, Clinical Modification (ICD-9-CM).

We first filtered the records of women who delivered in hospital using an ICD-9-CM code beginning with V27 (Outcome of delivery) and then analyzed this subset of data. The Texas PUDF contains 360,943 patients who delivered at the hospital. Of those, 14,375 (3.98%) developed PE. Table 2 summarizes the demographic attributes, such as age, race, ethnicity, insurance type, and whether or not the patient lives in a county on the Mexican border [82]. The frequency of each feature’s values (percentage of the population) is provided in this table.

**Table 2:** Patient demographic attributes in the Texas PUDF, where AA: African American, NA: Native America, A/PI: Asian or Pacific Islander.

Feature	Value	Frequency
Ethnicity	Hispanic	150,031 (41.57%)
	Non-Hispanic	207,494 (57.49%)
Race	White	195,149 (54.07%)
	AA	41,168 (11.41%)
	NA	1,214 (0.34%)
	A/PI	13139 (3.64%)
	Other	109,395 (30.31%)
Border County	Yes	44,989 (12.46%)
	No	315,954 (87.54%)
Insurance	Medicaid	185,010 (51.25%)
	Medicare	2,543 (0.70%)
	Self-pay/Charity	31,903 (8.84%)
	Other	176,312 (48.84%)
Age (years)	10–14	505 (0.14%)
	15–17	11,120 (3.08%)
	18–19	24,317 (6.74%)
	20–24	91,287 (25.29%)
	25–29	101,109 (28.01%)
	30–34	84,728 (23.47%)
	35–39	38,760 (10.74%)
	40–44	8,593 (2.38%)
	45–49	484 (0.13%)
	50–54	40 (0.01%)

**Fig 2:**
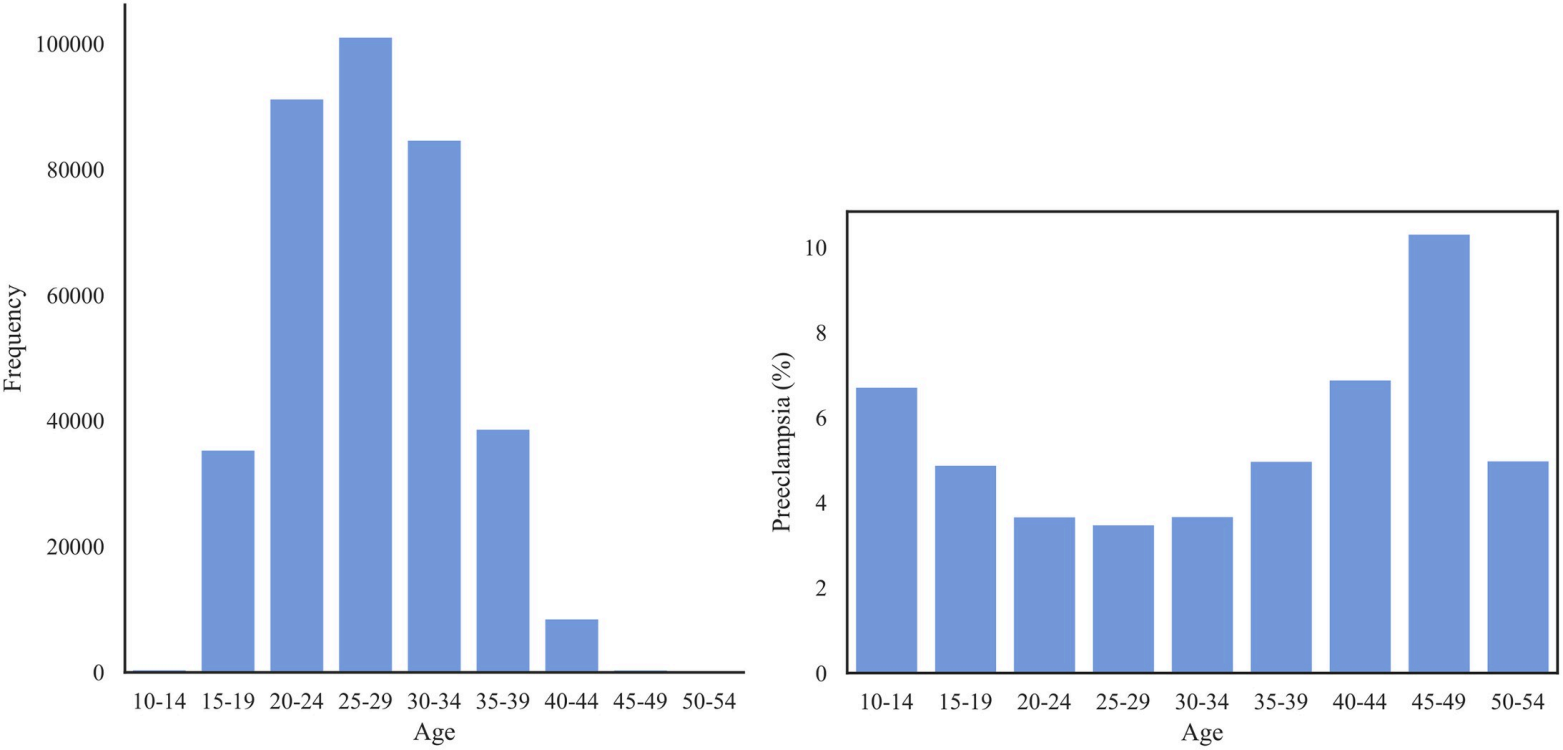
Left: The distribution of age group in Texas PUDF; Right: The prevalence of PE (%) for each age group.

Fig 2 shows that the majority of the patients lie within age range of 20 and 34. The prevalence of PE across age groups shows a U-shaped distribution with the most at-risk patients in the range 45–49, followed by patients of ages 40–44 and 10–14.

**Fig 3:**
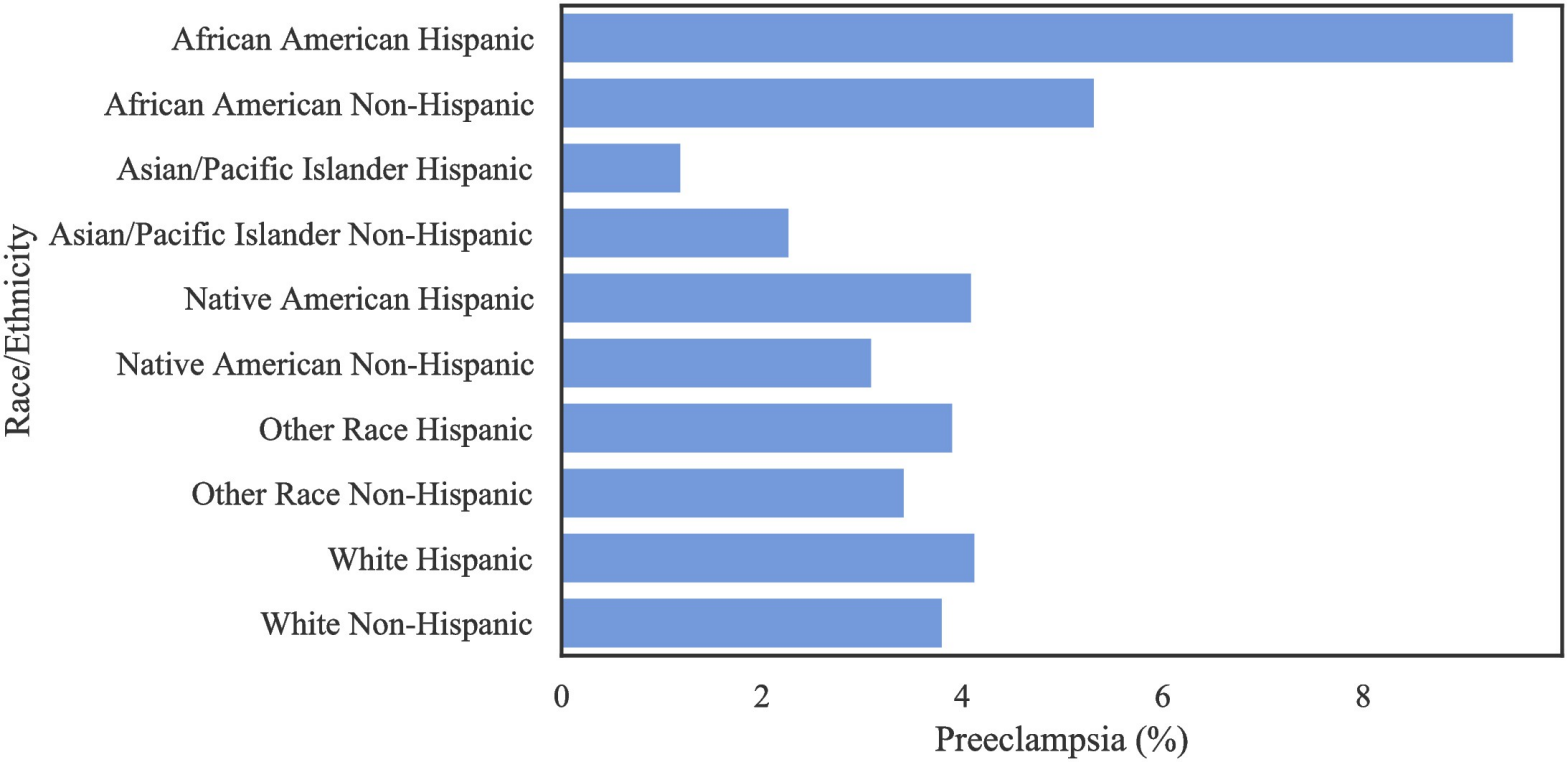
The rate of PE per race/ethnicity in the Texas PUDF.

**Fig 4:**
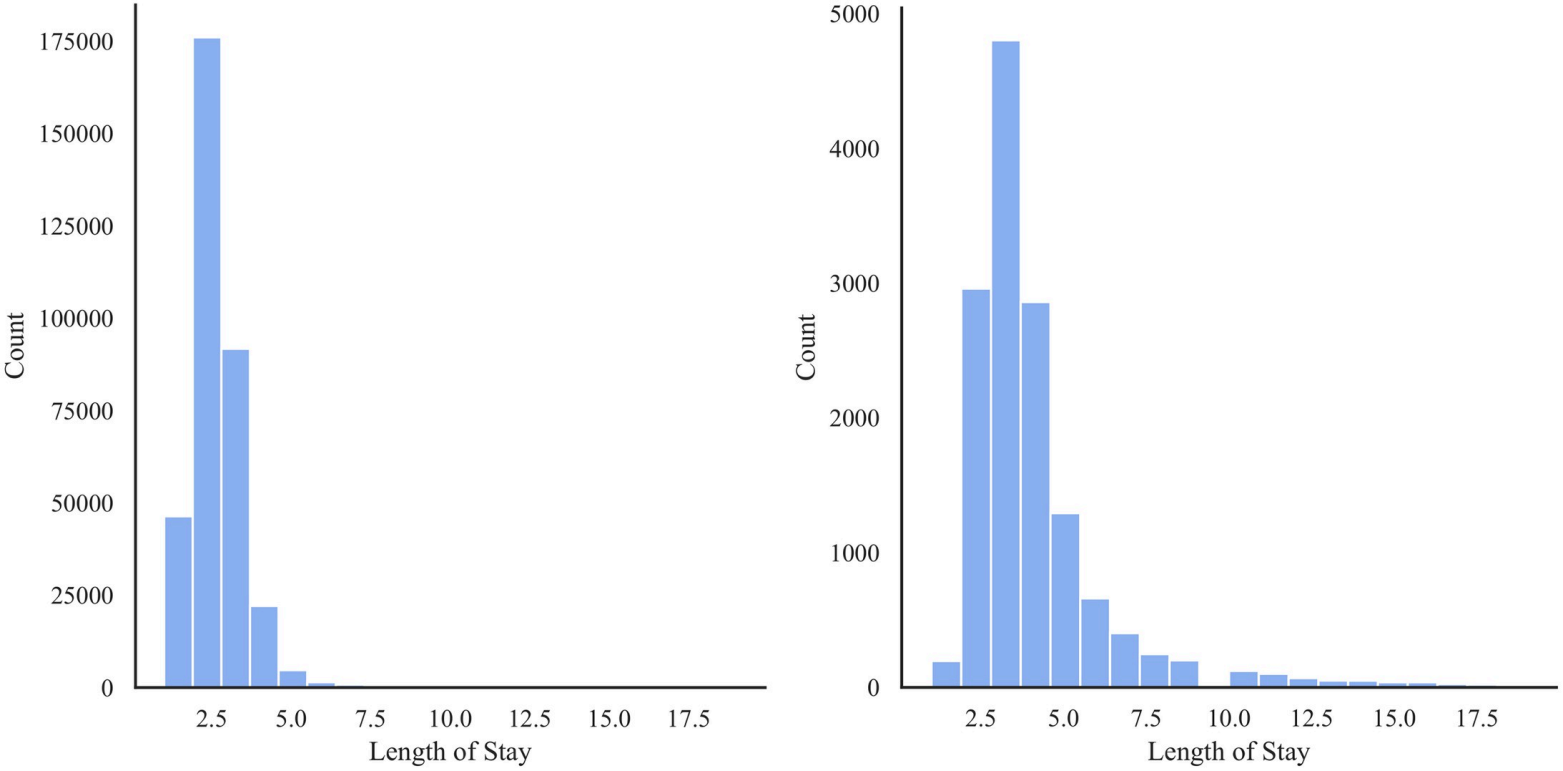
Distribution of length of stay for the Texas PUDF—Left: Women without PE; Right: Women with PE.

Table 3 shows the distribution of patients based on ethnicity and race. There is a large number of missing values regarding race among the non-Hispanic population, and a large amount of missing values regarding ethnicity amongst Other Races and the White population in this data. Table 4 and Fig 3 show the distribution of occurrence of PE by race. Notably, Hispanic AA patients had a higher incidence of PE, with a frequency of 9.51% (as a proportion of population) among all race/ethnic groups. Fig 4 shows that the preeclamptic women are more likely to have prolonged length of stay in hospital compared to Non-PE patients. In particular, AA Hispanic patients with PE have longer average stay in the hospital (Table 5).

**Table 3:** Distribution of the Texas PUDF [frequency (percentage)] based on race and ethnicity attributes, where AA: African American, NA:Native America, A/PI: Asian or Pacific Islander.

Race/Ethnicity	Hispanic	Non-Hispanic	Missing
AA	1,125 (0.027%)	39,743 (0.965%)	300 (0.007%)
NA	390 (0.321%)	805 (0.663%)	19 (0.016%)
A/PI	333 (0.025%)	12,676 (0.965%)	130 (0.010%)
White	59,500 (0.305%)	134,246 (0.688%)	1,403 (0.007%)
Other	88,505 (0.809%)	19,384 (0.177%)	1,506 (0.014%)
Missing	178 (0.203%)	640 (0.729%)	60 (0.068%)

**Table 4:** Distribution of preeclamptic patients among different race (AA: African American, NA: Native America, A/PI: Asian or Pacific Islander), and ethnic (Hispanic and non-Hispanic) groups in the Texas PUDF.

Race	Ethnicity	Preeclamptic
White	Hispanic	2461 (4.14%)
	Non-Hispanic	5117 (3.81%)
AA	Hispanic	107 (9.51%)
	Non-Hispanic	2118 (5.33%)
NA	Hispanic	16 (4.10%)
	Non-Hispanic	25 (3.10%)
A/PI	Hispanic	4(1.20%)
	Non-Hispanic	289 (2.28%)
Other	Hispanic	3464 (3.91%)
	Non-Hispanic	665 (3.431%)

**Table 5:** Length of stay (days) by race/ethnicity for patients with PE for the Texas PUDF. We report the average (Avg), standard deviation (SD), minimum (Min), first quartile (Q1), median, third quartile (Q3), and maximum (Max) values.

Race, Ethnicity	Avg	SD	Min	Q1	Median	Q3	Max
AA, Hispanic	**5.8**	4.8	2.0	3.0	**4.0**	6.0	37.0
AA, Non-Hispanic	5.0	6.0	1.0	3.0	**4.0**	5.0	107.0
A/PI, Hispanic	3.5	1.7	2.0	2.8	3.0	3.8	6.0
A/PI, Non-Hispanic	5.0	5.7	1.0	3.0	3.0	5.0	58.0
NA, Hispanic	4.3	3.7	1.0	2.0	3.0	4.3	13.0
NA, Non-Hispanic	4.0	3.8	1.0	2.0	3.0	4.0	21.0
Other Race, Hispanic	4.1	4.4	1.0	2.0	3.0	4.0	93.0
Other Race, Non-Hispanic	4.2	3.9	1.0	3.0	3.0	4.0	37.0
White, Hispanic	3.8	3.0	1.0	2.0	3.0	4.0	44.0
White, Non-Hispanic	4.6	4.7	1.0	3.0	3.0	5.0	105.0

The average length of stay for women with PE is longer. The median and interquartile range (IQR) of women with PE are 3 days and 1 day in comparison to the median and IQR of 2 days and 1 day for women without PE.

#### Case 2: Oklahoma PUDF

The second set of data that we use is the 2017 and 2018 Oklahoma Discharge Public Use Data Files (PUDF). The Oklahoma PUDF consists of statewide discharge data collected from two data sources: 1) the Uniform Claims and Billing Form (UB-92) for the hospital inpatient and outpatient surgeries, 2) the HCFA/CMS 1500 claims forms for the ambulatory surgery centers. It is maintained by the Health Care Information Division of the Oklahoma State Department of Health.

Unlike the 2013 Texas PUDF, the 2017–2018 Oklahoma PUDF used the ICD-10-CM diagnosis codes [83]. The women who delivered in hospital are filtered based on the ICD-10-CM codes beginning with Z37 (Outcome of delivery). This dataset contains a total of 84,632 women who delivered in-hospital, of which 4,721 (5.58%) developed PE. Table 6 demonstrates the demographic attributes, such as age, race, insurance type, and month of delivery. The frequency of each feature’s values (percentage of the population) is reported in this table. Unlike the Texas PUDF, no data on ethnicity is collected for each patient, but race remains an available variable along with additional attributes such as marital status and month of admission. There are no records indicating the delivery date for each patient in this data. We used the month of admission to estimate the month of delivery for each patient.

**Table 6:** Patient demographic attributes in the Oklahoma PUDF, where AA: African American, NA: Native America.

Feature	Value	Frequency	Feature	Value	Frequency
Race	White	55,815 (65.950%)	Month of Delivery	Jan	7,148 (8.446%)
	AA	8,510 (10.055%)		Feb	6,418 (7.583%)
	NA	5,443 (6.431%)		Mar	6,947 (8.208%)
	Other	14,864 (17.563%)		Apr	6,537 (7.724%)
Marital Status	Married	37,038 (43.760%)		May	7,242 (8.557%)
	Not Married	32,579 (38.490%)		Jun	7,031 (8.308%)
	Unknown	15,015 (17.740%)		Jul	7299 (8.624%)
Age Group	10–14	71 (0.084%)		Aug	7,699 (9.097%)
	15–19	6,192 (7.316%)		Sep	7,183 (8.487%)
	20–24	21,831 (25.795%)		Oct	7,371 (8.709%)
	25–29	26,708 (31.559%)		Nov	6,872 (8.120%)
	30–34	20,115 (23.768%)		Dec	6,885 (8.135%)
	35–39	8,164 (9.646%)			
	40–44	1,458 (1.723%)			
	45–49	84 (0.099%)			
	50–54	9 (0.011%)			
Insurance	Medicaid	42,192 (0.499%)			
	Medicare	450 (0.005%)			
	Self-pay	916 (0.011%)			
	Other Insurance	41,071 (0.485%)			

**Fig 5:**
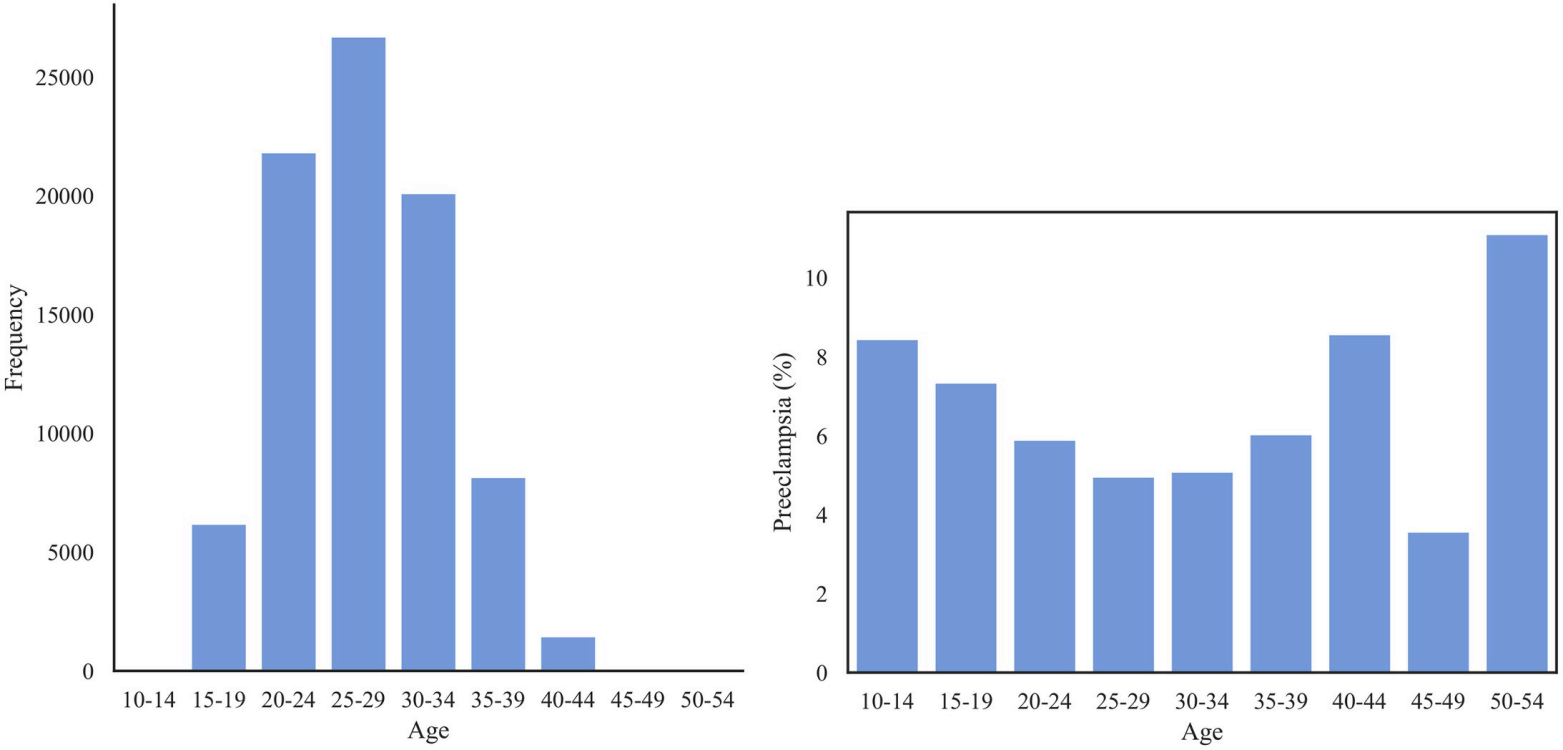
Left: The distribution of age group in the Oklahoma PUDF—Right: The prevalence of PE (%) for each age group.

Fig 5 shows the distribution of patients’ age groups and the prevalence of preeclampsia among them. Similar to the Texas PUDF, most of the patients are in the age range of 20–34. The prevalence represents a U-shaped curve highlighting the youngest and oldest patients as the most at-risk patients.

**Fig 6:**
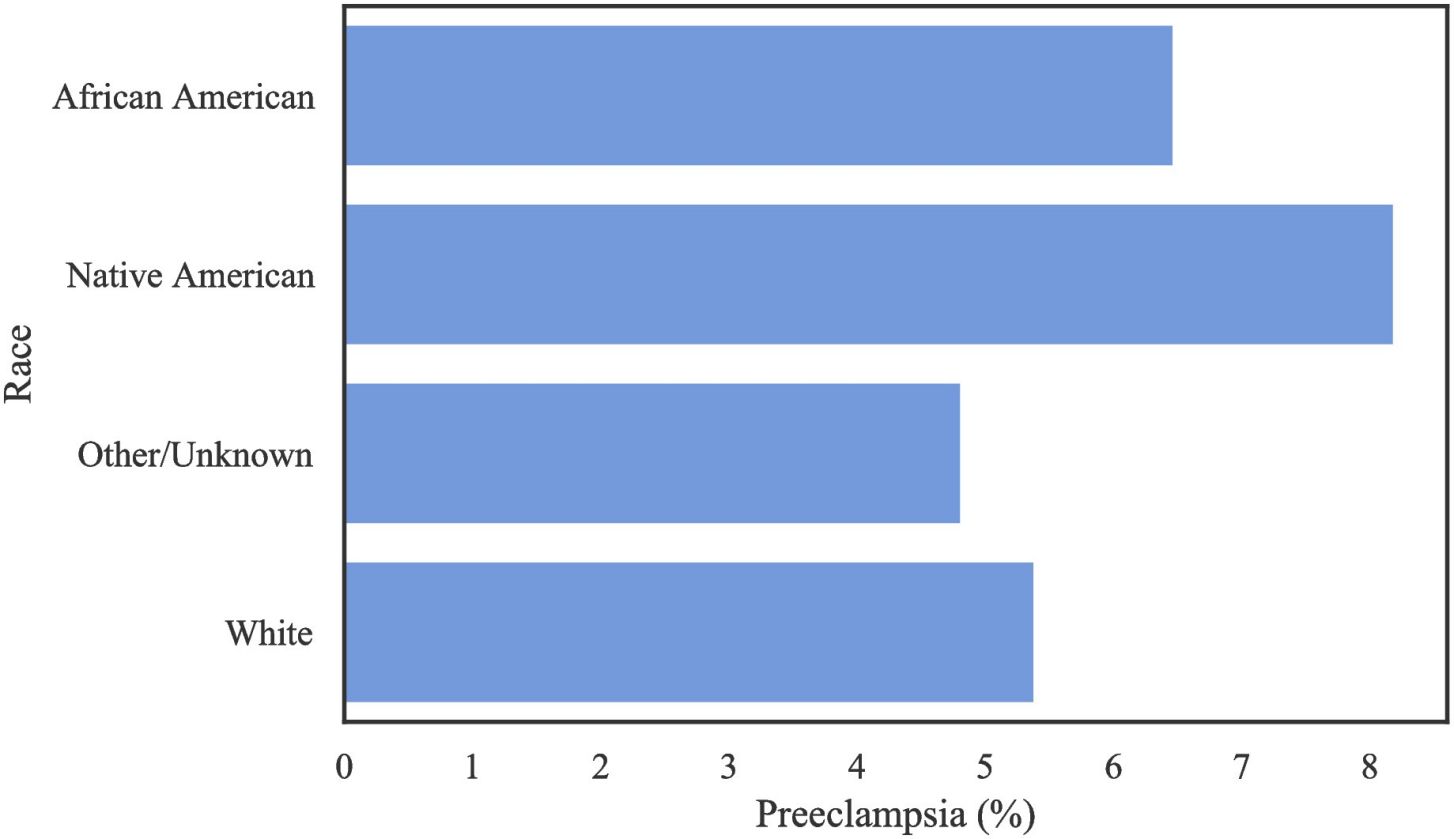
The rate of PE per race in the Oklahoma PUDF.

Table 7 shows the frequency of PE among each racial group in the Oklahoma PUDF, while Fig 6 shows the absolute number of patients in each racial category and their respective prevalence of PE. Despite White patients contributing the overwhelming majority of patients in this dataset, Native Americans and African Americans have the highest prevalence of PE. In particular, PE among Native Americans is almost twice that of the “Other/Unknown” race.

**Table 7:** Distribution of preeclamptic patients among different race in the Oklahoma PUDF, where AA: African American, NA: Native American.

Race	Preeclamptic
White	3008 (5.39%)
AA	551 (6.57%)
NA	446 (8.19%)
Other/Unknown	716 (4.82%)

**Fig 7:**
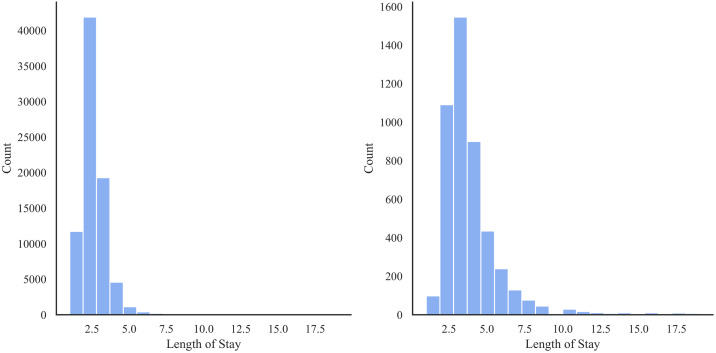
Distribution of length of stay—Left: Women without PE; Right: Women with PE.

Similar to the Texas PUDF, the average length of stay is longer for patients with PE compared to those without PE as shown in Fig 7. The median and IQR length of stay for those without PE is 2 and 1 days respectively, while for those with PE, the median and IQR length of stay is 3 and 2 days respectively.

The length of stay also varies with a patient’s race. According to the Table 8, we notice that AAs and NAs had longer hospital stays compared to their white and “other” counterparts.

**Table 8:** Length of stay by race for patients with PE in Oklahoma PUDF. We report the average (Avg), standard deviation (SD), minimum (Min), first quartile (Q1), median, third quartile (Q3), and maximum (Max) values.

Race	Avg	SD	Min	Q1	Median	Q3	Max
AA	4.2	3.3	1.0	3.0	3.0	5.0	35.0
NA	**4.5**	4.4	1.0	3.0	3.0	5.0	57.0
Other/Unknown	3.8	3.7	1.0	2.0	3.0	4.0	57.0
White	4.0	3.9	1.0	2.0	3.0	4.0	84.0

After selecting the initial set of clinical features, each was formulated as a binary feature based on the presence of corresponding ICD-9-CM/ICD-10-CM codes in any of the diagnosis columns. We set the value of the feature equal to 1 if the corresponding ICD-9-CM/ICD-10-CM codes used in this study were present in any of the diagnosis columns; otherwise the value was set to zero. A full list of ICD-9-CM/ICD-10-CM codes is described in the S3 Table of the S1 File. Furthermore, a detailed analysis of prevalance of each code in both Texas and Oklahoma datasets can be found in the S5 Table of the S1 File.

#### Case 3: MOMI

Maintained by University of Pittsburgh’s Medical Center Magee-Womens Hospital since 1995, the MOMI Database reports about 300 variables for more than 200,000 deliveries. The dataset is extracted from medical records coding, admitting services, outpatient encounters, ultrasound, and other ancillary systems for all mother-infant pairs delivered at Magee. Unlike the Texas and Oklahoma data, this dataset contains patient information from multiple prenatal visits in addition to the demographic and clinical features. For this study, we considered patients’ information in their latest prenatal visit within the first 14 weeks, which includes in total 31,431 women who delivered in-hospital, of which 2,743 (8.73%) developed PE. The demographic attributes along with their frequency (percentage of the population) are represented in Table 9. The clinical attributes of the MOMI data are summarized in the S6 Table of the S1 File.

**Table 9:** Patient demographic attributes in the MOMI data. We report the average (Avg), standard deviation (SD), minimum (Min), and maximum (Max) values for numeric non-discrete attributes.

Feature	Value	Frequency	Feature	Statistic	Value
Race	Polynesian	36 (0.12%)	Mother’s Age	Avg	30.13
	NA	102 (0.33%)		SD	5.33
	White	22,184 (70.58%)		Min	13
	Asian	2,108 (6.71%)		Max	52
	AA	6,354 (20.22%)	Weight at Admission	Avg	86.28
	Missing	647 (2.06%)		SD	19.14
Insurance	Self-pay	378 (1.20%)		Min	30.58
Classification	Medicare/Medicaid	10,336 (32.89%)		Max	259
	Private Insurance	20,710 (65.89%)	Infant Number	Avg	1.57
Infant Sex	Female	15,003 (47.73%)		SD	0.86
	Male	16,012 (50.94%)		Min	1
				Max	10
			Prenatal Weight	Avg	2,616.58
				SD	694.64
				Min	176
				Max	7,456

Furthermore, we added a new feature for each patient which represented the number of incidents of spikes in blood pressure within the first 14 weeks. A spike in blood pressure is defined as systolic pressure above 130 or diastolic pressure above 80. In addition, this dataset consists of several numeric variables, including weight, age, previous pregnancies, the number of abortions and deliveries, etc. All numeric variables are normalized so that all values are within the range of 0 and 1 prior to training ML models. The race variable for patients from the groups “Indian(Asian)”, “Chinese”, “Korean”, “Filipino”, “Japanese”, “Vietnamese”, and “Other Asian” are labeled as “Asian.” We noted that patients from any of the cohorts “Hawaiian”, “Samoan”, “Guam/Chamorro”, and “Other Pacific Islander” are identified as “Polynesian” race. We include “Native American” and “Alaskan Native” patients as one group (“Native American (NA)”). Ethnicity (whether or not a mother is Hispanic) is included as a variable in the MOMI dataset, however 42% of this variable was missing, and as such we dropped this variable from our analysis.

**Fig 8:**
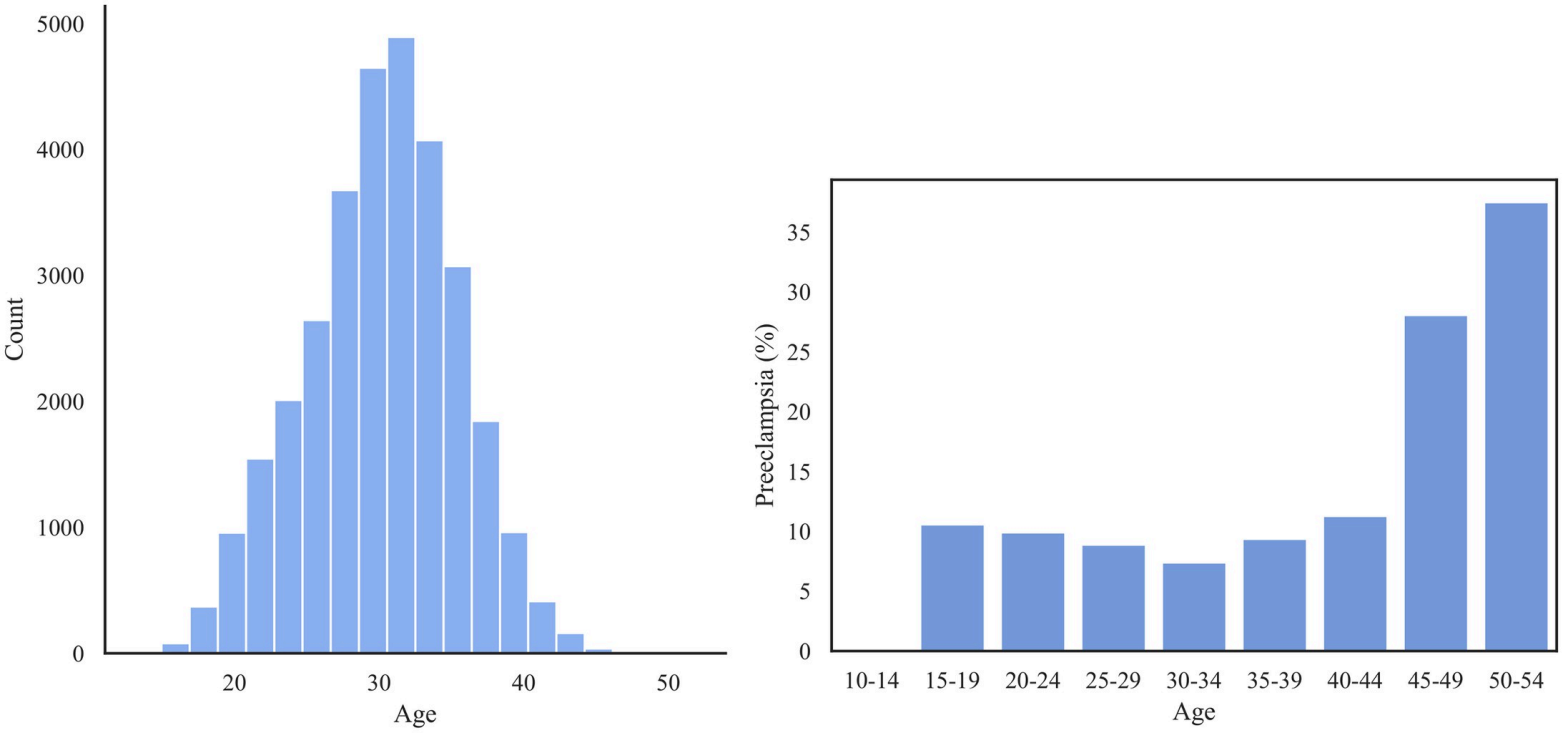
Left: The distribution of age group in the MOMI dataset; Right: The prevalence of PE (%) for each age group.

Fig 8 shows the prevalence of PE within the different age groups. We observed that there is a noticeable U-shaped trend in incidence which reflects the high risk of PE among the oldest patients and a slightly increased risk among the youngest patients.

**Fig 9:**
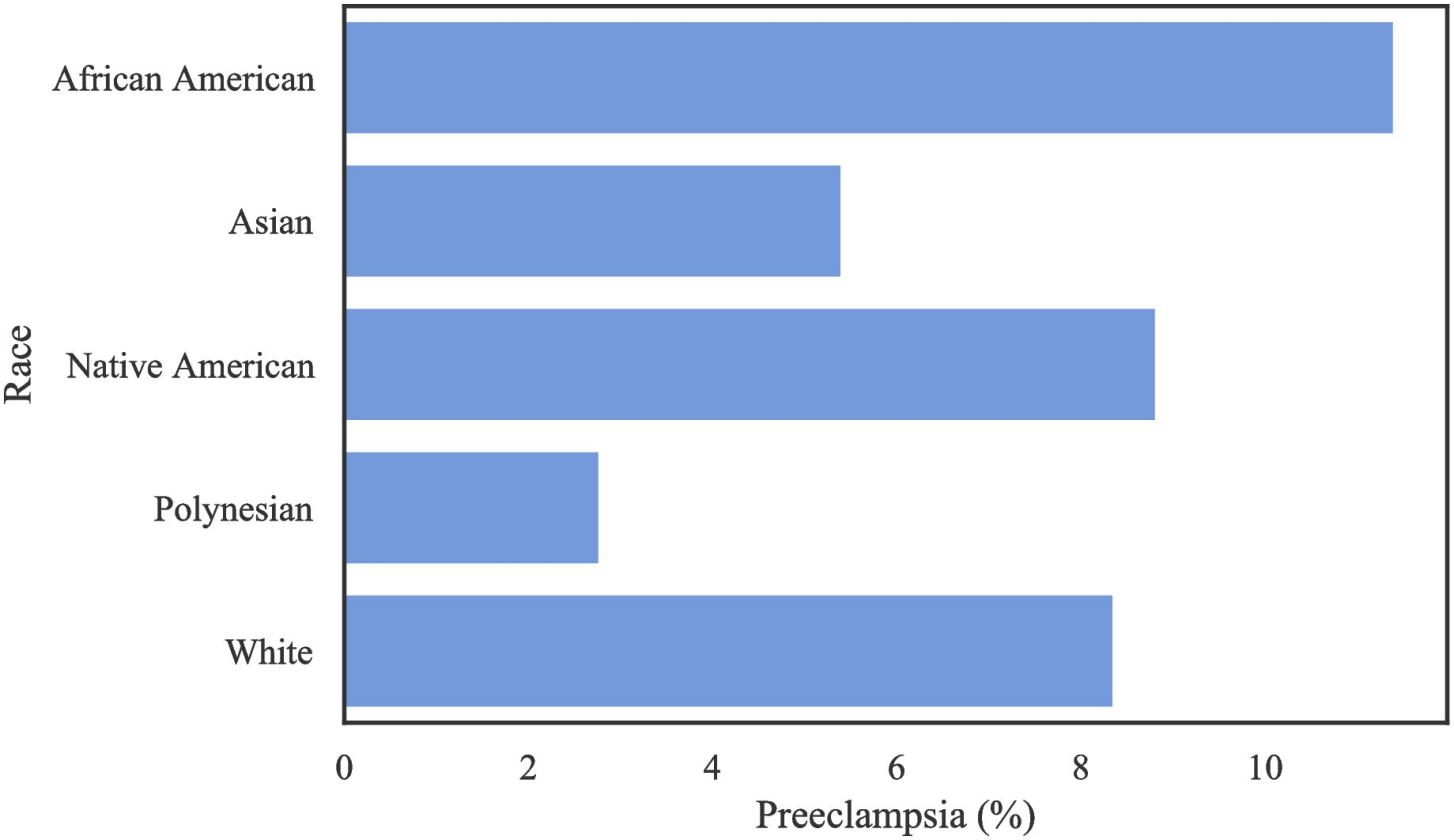
The rate of PE per race in the MOMI.

Fig 9 represents the prevalence of PE among different racial groups in MOMI dataset. African American patients experience a higher rate of PE (11.4%) compared to other racial groups.

To reduce the computational complexity and increase the interpretability of our results, we used Chi-square feature selection to extract the most critical variables related to preeclampsia, which will be described in the Results Section.

#### Missing data challenge

In the Texas PUDF set, the county feature has the most significant number of missing values (2.5%), followed by the patients’ ethnicity (0.95%), race (0.24%), and insurance (0.04%). In the Oklahoma PUDF, the most common missing values was marital status variable (17.74%), followed by the county (0.004%) and insurance (0.004%) variables.

In the MOMI dataset, features with greater than 20% missing values were dropped prior to any preprocessing steps. The most frequently missing feature is race (2.06%), followed by the mean arterial pressure (1.40%) and infant sex (1.32%). Furthermore, we observed that if there was a missing value for the total number of previous pregnancies for a patient, some others such as deliveries, miscarriages, abortions, and whether or not this is a first pregnancy were also missing. More details about features with missing values are provided in the S7-S9 Tables of the S1 File. We used a Multiple Imputation technique [84] to estimate missing values. For the Multiple Imputation implementation, we used Bayesian ridge regression [85] repeated 10 times in order to gain a more robust estimate for the final imputed values.

## Results

### Feature selection

Using Chi-square feature selection, we identified the 20 most important risk factors which are ranked in terms of variable importance for each of the three datasets.

#### Case 1: Texas PUDF

**Fig 10:**
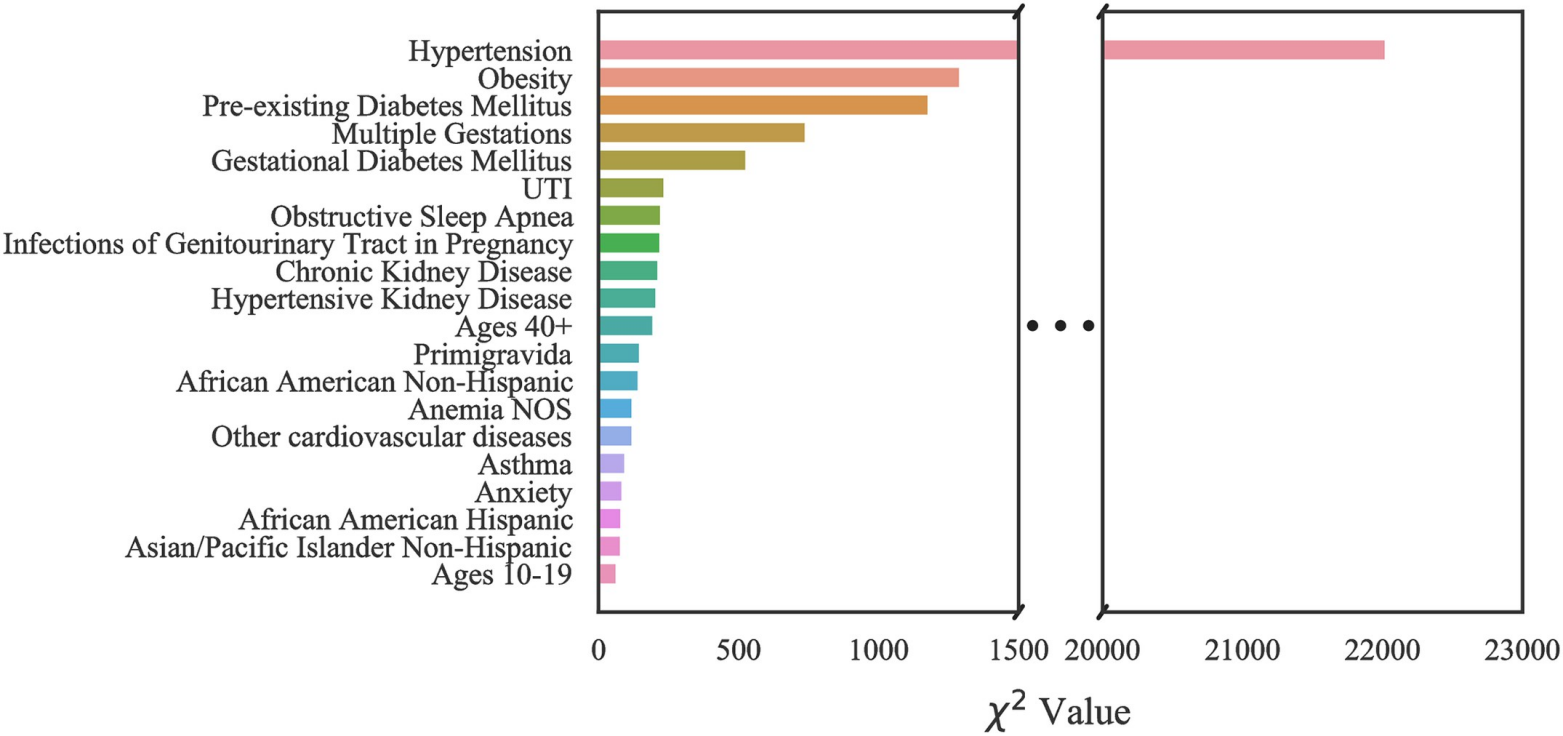
Feature ranking for the Full Texas dataset.

**Fig 11:**
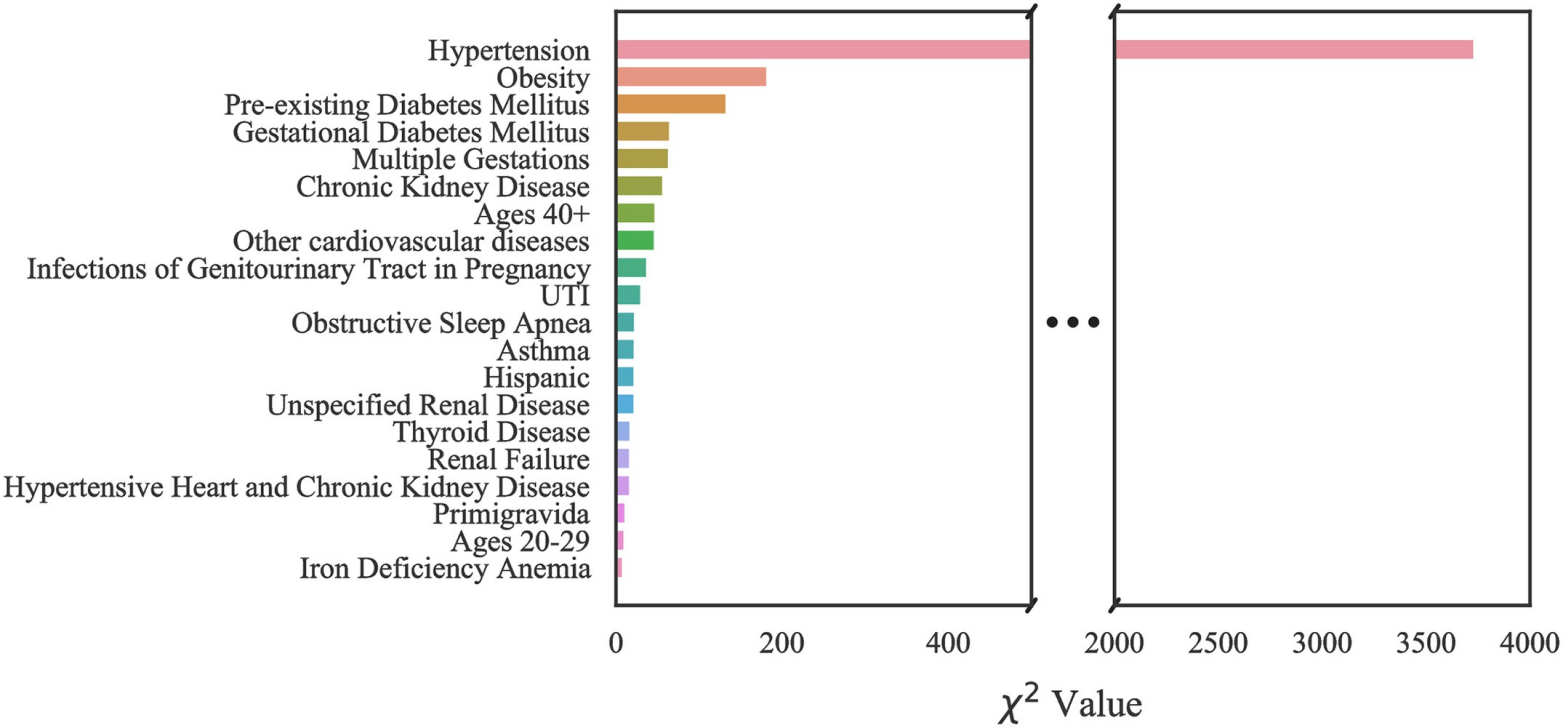
Feature ranking for the Texas African American dataset.

**Fig 12:**
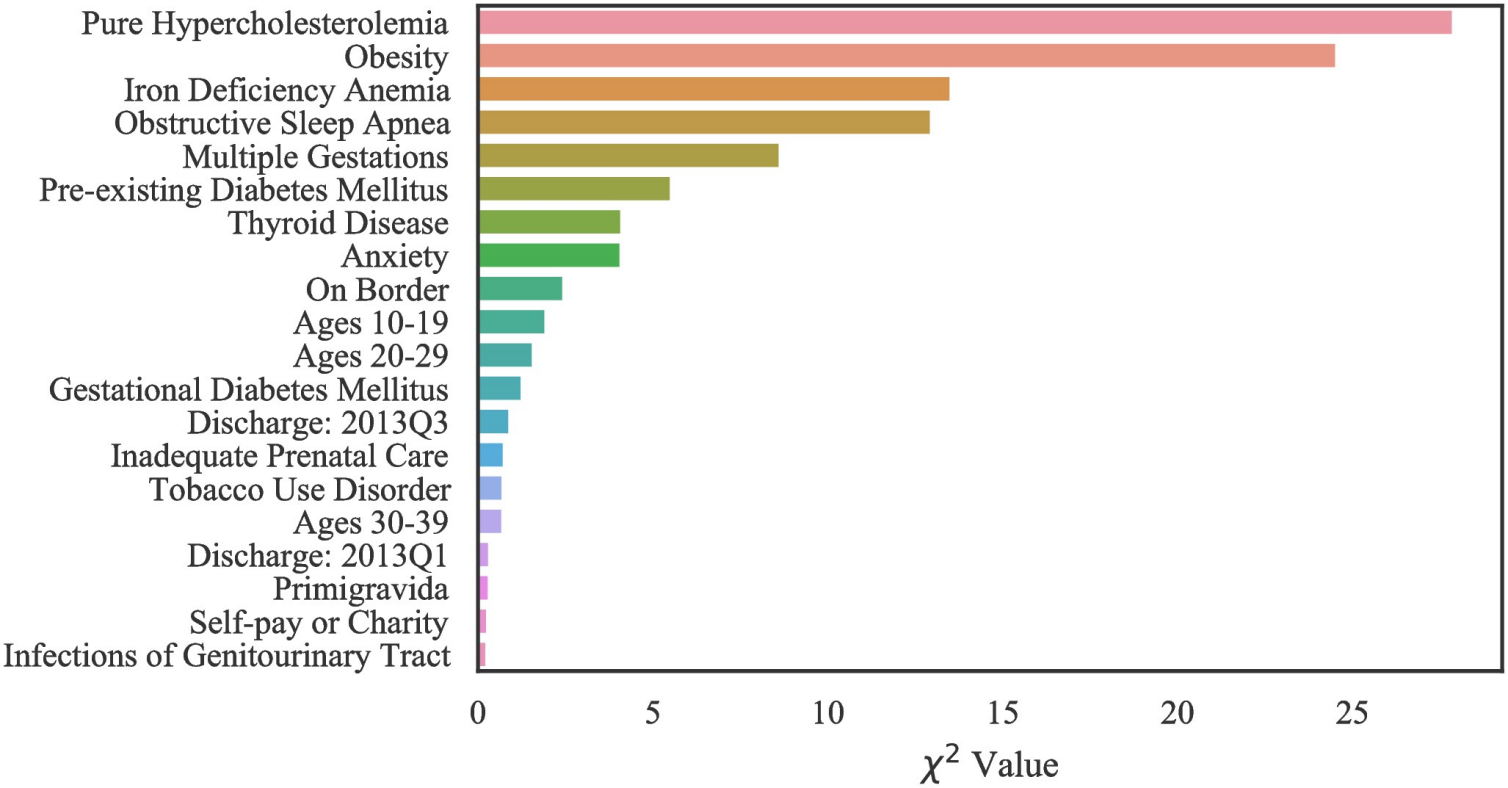
Feature ranking for the Texas Native American dataset.

The top 20 critical risk factors related to Texas PUDF general (full) population as well as AA and NA populations are represented in Figs 10–12. Although there are differences in which features are chosen among the various groups, there is a considerable overlap among them. For example, obesity is the highest ranked feature in the full, only NA, and only AA population datasets. In the AA dataset, there are six features that are indicated as important based on Fig 11, but they do not appear in the full population’s most important features. These are unspecified renal disease, thyroid disease, renal failure, hypertensive heart and chronic kidney disease, ages 20–29, and iron deficiency anemia.

Fig 12, indicates that amongst NA patients, there are a greater number of features that do not overlap with the general population. These features are pure hypercholesterolemia, iron deficiency anemia, thyroid disease, location on the Mexican border, ages 20–29, discharge/delivery in the third quarter, inadequate prenatal care, tobacco use disorder, ages 30–39, discarge/delivery in the first quarter, primigravida, and self-pay or charity with respect to payment methods.

#### Case 2: Oklahoma PUDF

**Fig 13:**
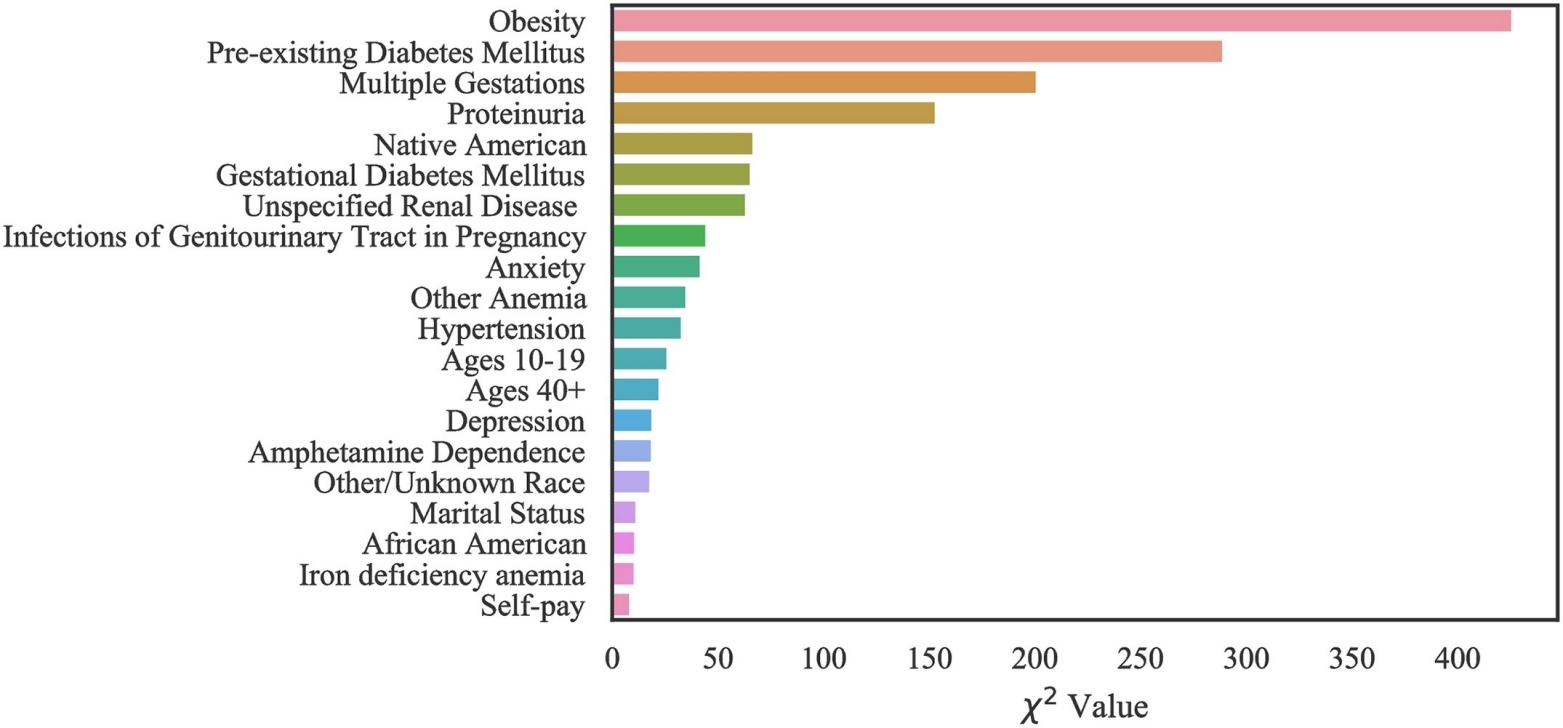
Feature ranking for the Full Oklahoma dataset.

**Fig 14:**
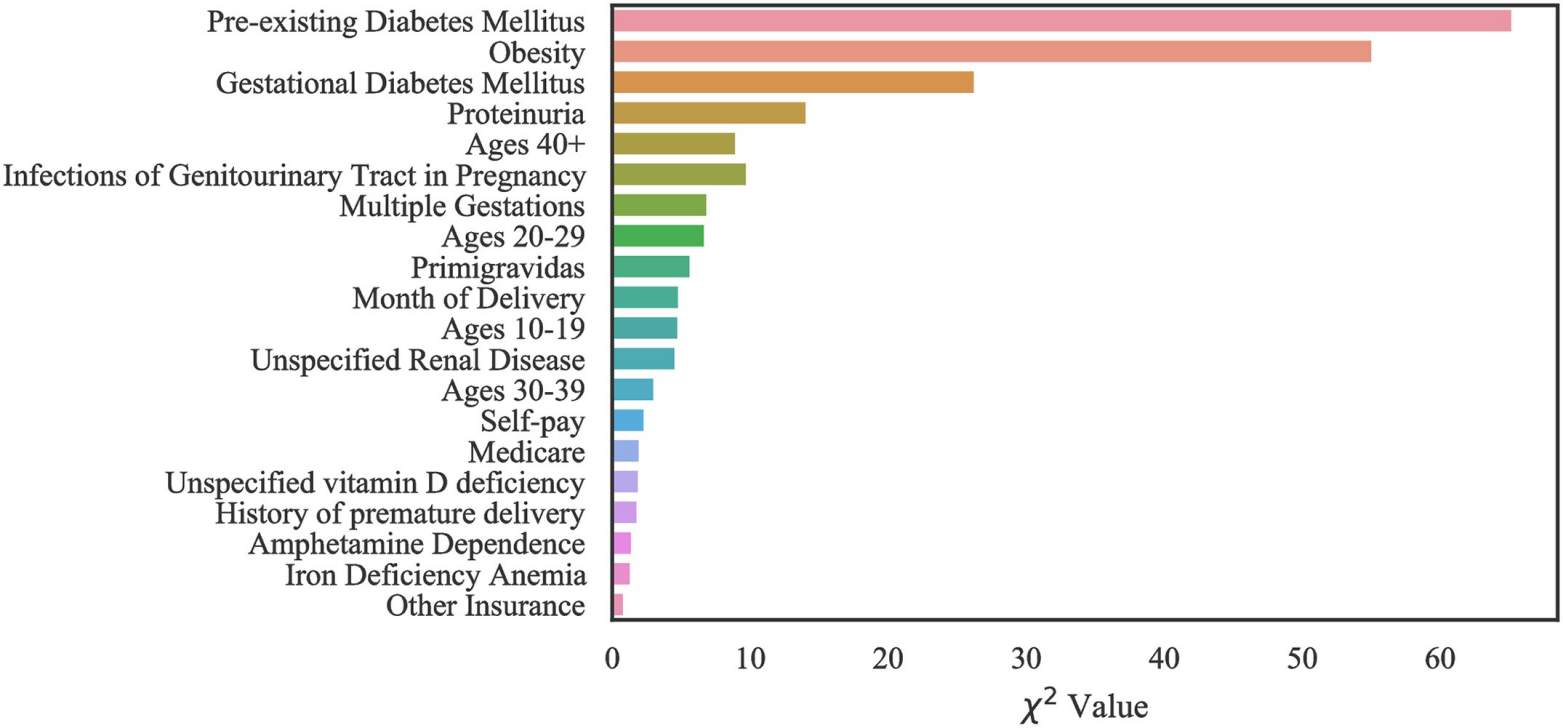
Feature ranking for the Oklahoma African American dataset.

**Fig 15:**
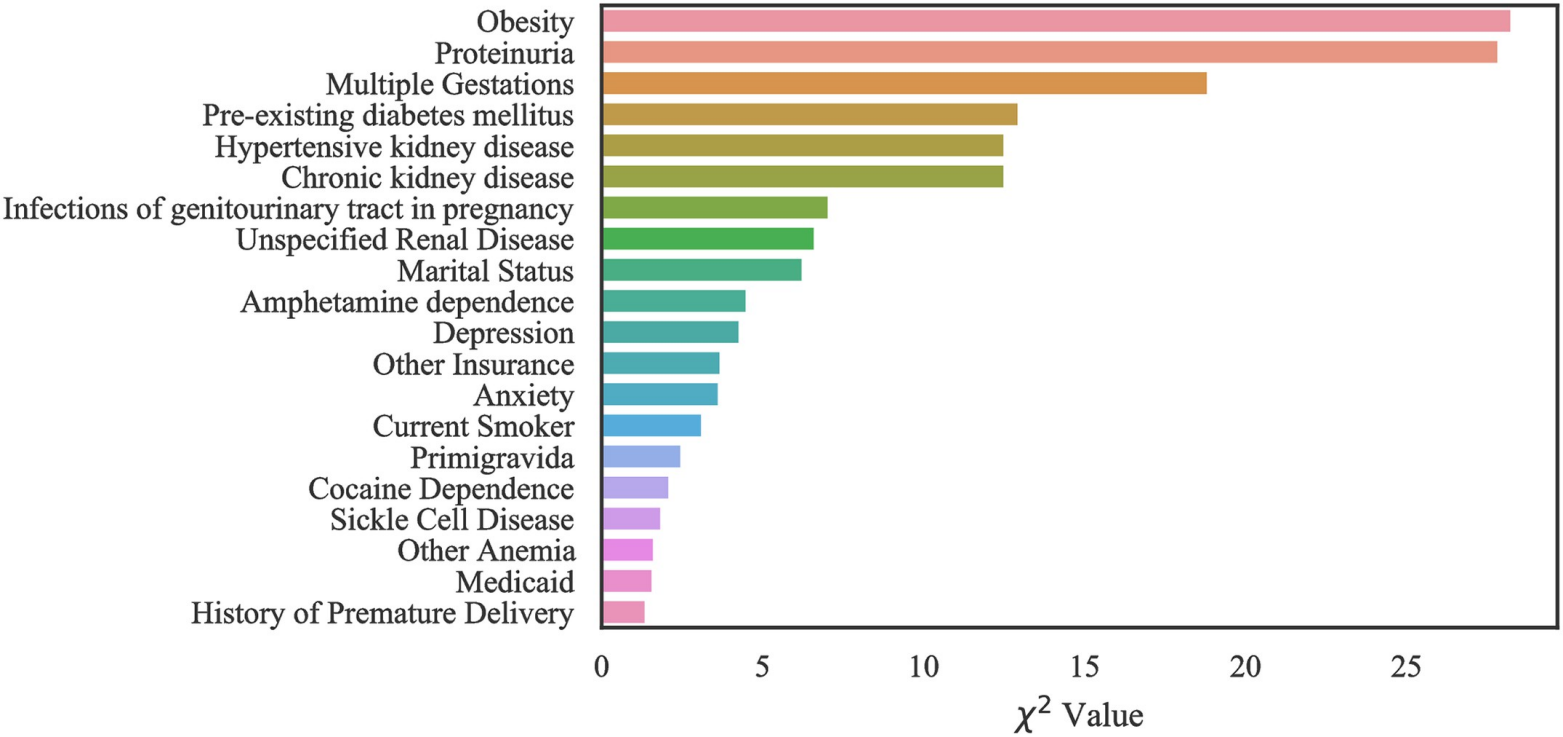
Feature ranking for the Oklahoma Native American dataset.

Figs 13–15 show the ranked top 20 risk factors related to Oklahoma PUDF general (full) population as well as AA and NA populations. Although there are differences in which features are chosen among the various groups, there is also a considerable overlap. For example, obesity is the highest, second highest, and third highest ranked feature in the full, African American, and Native American populations datasets respectively. In the African American dataset as shown in the Fig 14, there are 7 features that are indicated as important and differ from the full population. These include primigravida, month of delivery, age range of 20–29, age range of 30–39, Medicare, unspecified vitamin D deficiency, and history of premature delivery. According to Fig 15, in the Native American dataset, there are a greater number of specific features that do not overlap with the general population. These features are hypertensive kidney disease, UTI, cocaine dependence, and history of premature delivery.

#### Case 3: MOMI data

**Fig 16:**
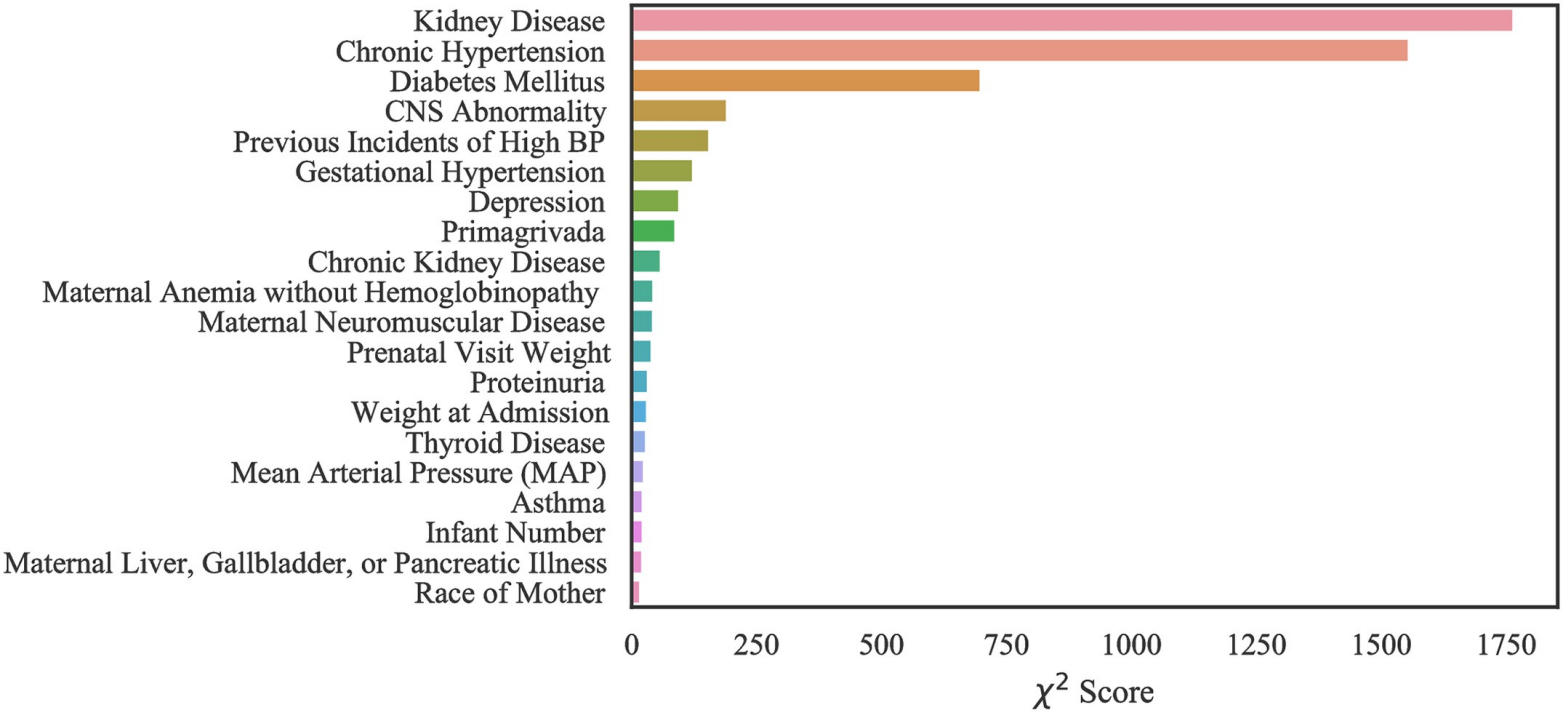
Feature ranking for the MOMI Full dataset.

**Fig 17:**
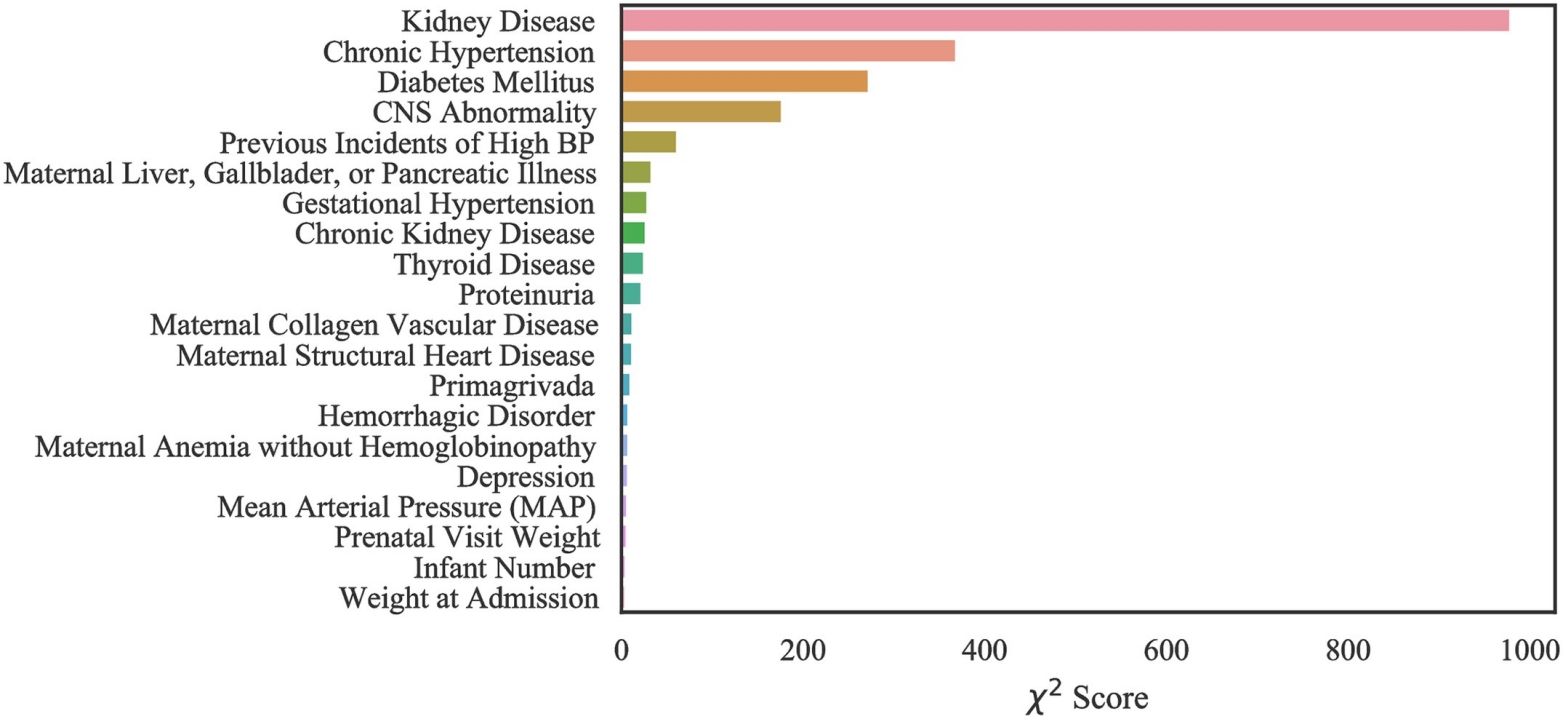
Feature ranking for the MOMI African American dataset.

Our computational results with MOMI data show that our models performed best when all features are considered in training. However, we identified the top 20 most important features in order to improve the interpretability of the data (Figs 16 and 17). Like the Texas and Oklahoma datasets, hypertension and diabetes are significant predictors, however other variables such as kidney disease are among the important risk factors which were not identified within the Texas and Oklahoma datasets (case 1 and 2). Interestingly, we note that the number of previous spikes in high blood pressure was among the significant risk factors.

In the African American dataset as shown in the Fig 17, there were four features that were identified as important and do not overlap with the full population. These include maternal collagen vascular disease, maternal structural heart disease, hemorrhagic disorders, and maternal anemia without hemoglobinopathy.

### Model evaluation and discussion

In this section, we present the results of DNN and the proposed CSDNN algorithms on Oklahoma, Texas, and MOMI datasets. The performance of these algorithms were compared based on the evaluation measures described in the Performance Measures Section. We implemented both DNN and CSDNN algorithms in Python version 3.6 with Keras [86] and TensorFlow libraries [87]. All experiments and data processing were performed on an AMD Ryzen 5 3.8 GHz 6-Core processor and 16GB of Ram in a 64-bit platform. As a preprocessing step prior to classification, continuous variables were normalized such that they have zero mean and unitary standard deviation. In the MOMI dataset, outliers were removed using Local Outlier Factor [88]. Furthermore, a drop-out rate of 20% was applied to reduce the risk of overfitting for training both DNN and CSDNN.

#### CSDNN architecture

Neural Networks contain many hyperparameters that are needed to be set prior training such as learning rate, depth, the number of nodes per layer, activation functions, and weight initialization strategy. Given the large number of hyperparameters, we have found the best performing combination of hyperparameters for both DNN and CSDNN models using three hyperparameter strategies: Random Search [89], Bayesian optimization [90], and Hyperband [91]. All hyperparameter tuning is performed using the Keras Tuner library [92].

The initial ranges of each hyperparameter for model selection algorithms are summarized in Table 10. These hyperparameters are the batch size (*B*), the number of epochs (*T*), the number of hidden layers (*h*), the number of neurons in hidden layers (*k*), the learning rate (*η*), and activation functions (*a*). Each model selection algorithm is performed on each dataset using 10-fold cross validation repeated 5 times to increase the robustness of results, while for small sub-population datasets we performed 10-fold cross validation repeated 35 times. The best set of hyperparameters was selected based on the model selection that yields the highest G-mean. The best architecture along with hyperparameters obtained from the three model selection techniques for the best architecture of the DNN and CSDNN with WCE and FL functions as well as hybrid models that further balances batches with oversampling (Balanced Batches) are summarized in S10-S15 Table in S1 File. We observe that the Hyperband model selection consistently performs well on all datasets for both DNN and CSDNNs. The number of layers in most models does not exceed 4. Most models have employed larger learning rate (e.g., 0.001), but a few of the smaller datasets (e.g., Oklahoma NA datasets) have chosen smaller learning rates (0.00001), particularly for DNN and CSDNN-WCE that used the balanced batches method. Overfitting can be mitigated through early stopping of the neural networks. A detailed analysis of training and validation AUC versus the number of epochs for each model can be found in the S1-S6 Figs in S1 File.

**Table 10:** Summary of hyperparameter ranges for DNN and CSDNN.

	*B*	*T*	*k*	*h*	*η*
Range	64–8096	10–200	32–64	2–8	0.0001–0.01

#### Comparative analysis of CSDNN-FL versus parameters *γ* and *α*

**Fig 18:**
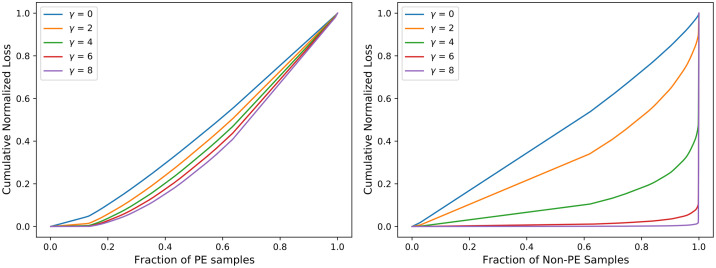
Cumulative distribution functions of the normalized loss for PE (positive) and Non-PE (negative) samples for various *γ* values for Texas PUDF and *α* = 0.5.

**Fig 19:**
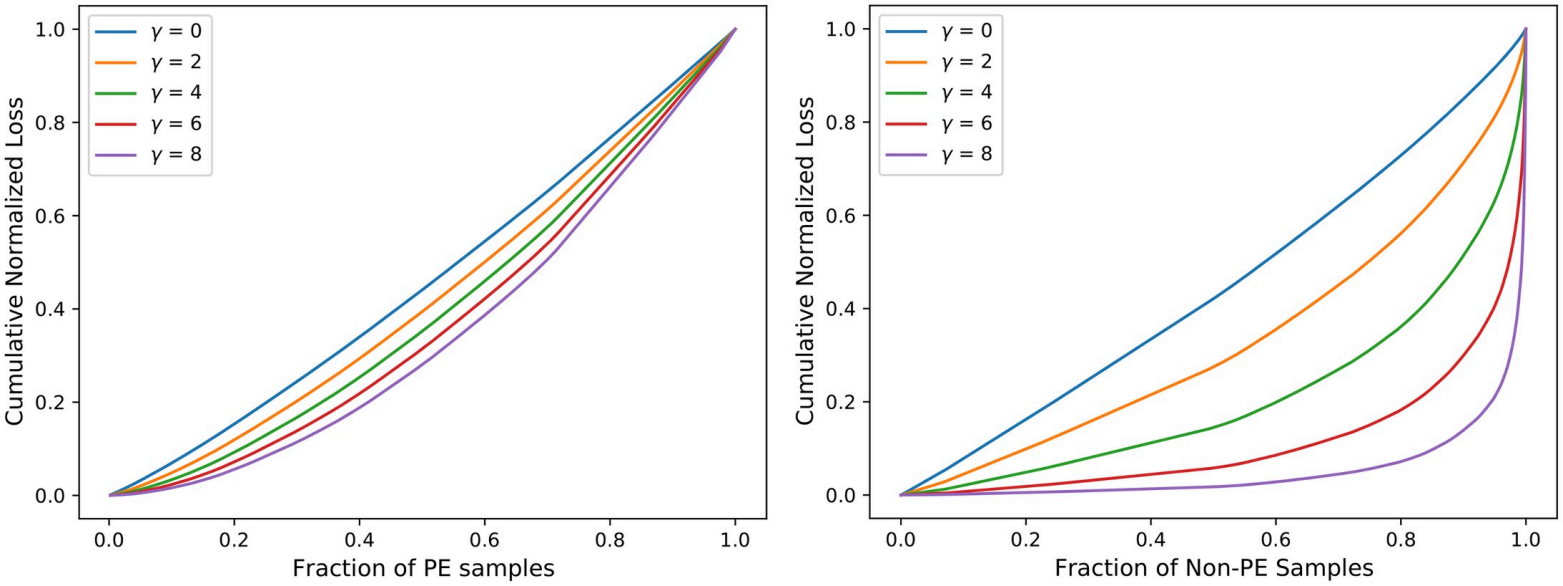
Cumulative distribution functions of the normalized loss PE (positive) and Non-PE (negative) samples for various *γ* values and *α* = 0.5 for Oklahoma PUDF.

**Fig 20:**
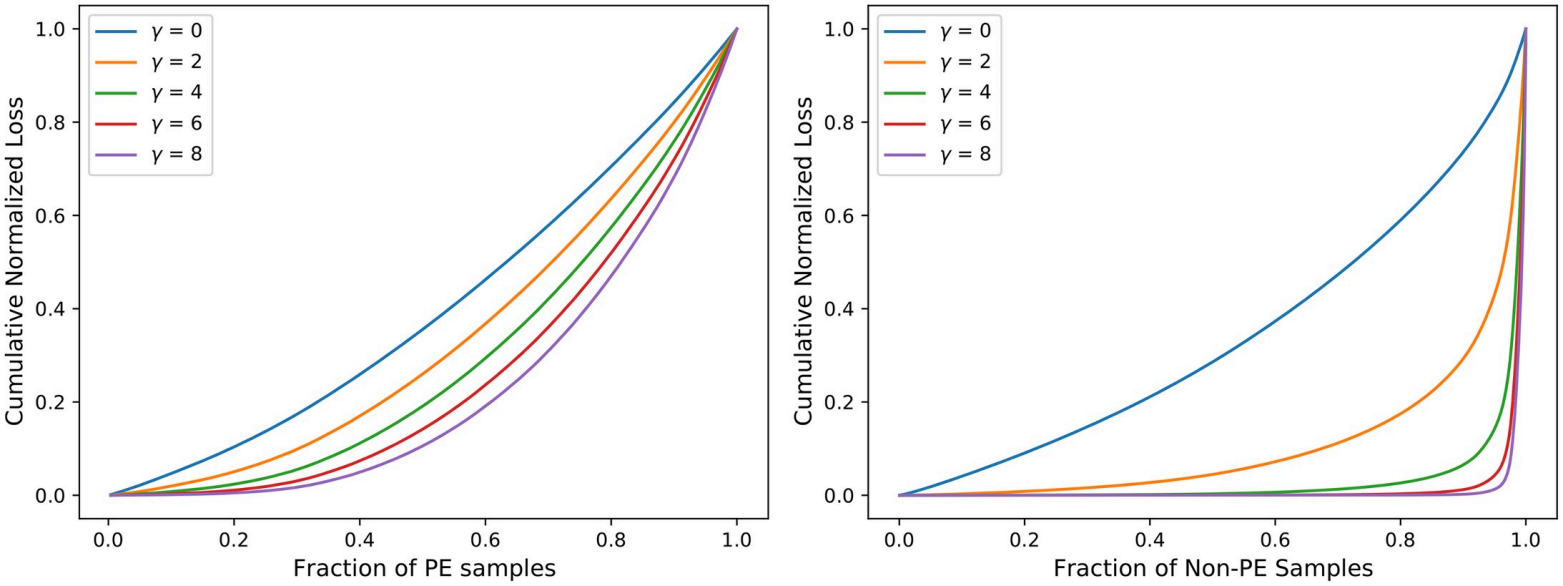
Cumulative distribution functions of the normalized loss for PE (positive) and Non-PE (negative) samples for various *γ* values and *α* = 0.5 for MOMI data.

To further investigate the effect of FL function on CSDNN performance, we obtained the cumulative loss generated from different values of the *γ* parameter. Inspired by the original paper by Lin et al. [47], Figs 18–20 were created by training the CSDNN-FL model with the best performing *α* for each dataset and different values of *γ*. The samples in the test set wer split into the positive and negative samples, and the loss is calculated for each sample using different values of *γ*. Then, the plots were created by ordering the normalized loss from lowest to highest and plotting the cumulative sums for the positive and negative classes for various *γ*, resulting in the cumulative sum plots (CSPs) shown in Figs 18–20. The effect of *γ* on positive samples (PE cases) was not as noticeable, however the effect of *γ* on negative samples (Non-PE cases) was substantially different. Both positive and negative CSPs appeared relatively analogous when *γ* = 0. By increasing the *γ* had a large effect on down-weighting the easy negative samples, as FL focuses learning on hard negative samples. This was consistent with earlier literature on FL [47].

The Tables 11–13 show that as *γ* increased, AUC decreased, but G-mean increases slightly except in the Texas dataset. CSDNN-FL for the Full Texas dataset showed relatively inferior specificity and higher recall values as *γ* increaseed. The results in these tables are obtained after calculating the best performing *α*, and then fixing *α* when calculating *γ*.

**Table 11:** Comparative analysis of CSDNN-FL versus *γ* using the Full Texas dataset. ACC, SP, PR, and RE represents accuracy, specificity, precision, and recall, respectively. The highest G-mean and AUC values are denoted in bold. The parameter *α* is set as 0.97.

*γ*	0	2	4	6	8
ACC	0.775	0.759	0.759	0.759	0.698
G-mean	**0.573**	0.561	0.560	0.561	0.515
AUC	**0.634**	**0.634**	0.633	0.633	0.633
SP	0.789	0.772	0.772	0.772	0.706
RE	0.438	0.449	0.449	0.450	0.497
PR	0.084	0.083	0.083	0.083	0.079

**Table 12:** Comparative analysis of CSDNN-FL versus *γ* using Oklahoma Full dataset. ACC, SP, PR, and RE represents accuracy, specificity, precision, and recall, respectively. The highest G-mean and AUC values are denoted in bold. The parameter *α* is set as 0.95.

*γ*	0	2	4	6	8
ACC	0.636	0.685	0.674	0.657	0.735
G-mean	0.603	0.613	0.611	**0.614**	0.593
AUC	**0.658**	**0.658**	0.650	0.648	0.647
SP	0.640	0.693	0.681	0.662	0.750
RE	0.568	0.542	0.549	0.570	0.468
PR	0.085	0.094	0.092	0.090	0.010

**Table 13:** Comparative analysis of CSDNN-FL versus *γ* using the MOMI Full dataset. ACC, SP, PR, and RE represents accuracy, specificity, precision, and recall, respectively. The highest G-mean and AUC values are denoted in bold. The parameter *α* is set as 0.92.

*γ*	0	2	4	6	8
ACC	0.801	0.810	0.751	0.747	0.723
G-mean	0.664	0.676	0.665	0.675	**0.682**
AUC	0.762	**0.765**	0.751	0.756	0.745
SP	0.826	0.835	0.768	0.761	0.732
RE	0.534	0.547	0.577	0.599	0.635
PR	0.227	0.240	0.192	0.193	0.185

#### Comparative analysis of csdnns with balanced batch method

We also compared our proposed CSDNN-FL and CSDNN-WCE with the standard DNN with and without Balanced Batches (BB) on the Full Texas, Oklahoma, and MOMI datasets as well as sub-population datasets. We reported the average G-mean, AUC, accuracy, precision, recall, and specificity values in Table 14. We observed that CSDNN-FL performs better compared to CSDNN-WCE and DNN on the Texas and Oklahoma datasets (in terms of G-mean and AUC). In Oklahoma African American dataset, we observed that there is no statistically significant difference between CSDNN-WCE and CSDNN-FL results. A detailed description is presented in Statistical Analysis Section. Interestingly, DNN and CSDNN-FL with balanced batches performed better than other methods for MOMI Full data and MOMI African American datasets, respectively. We also observed that in all cases the CSDNN-FL and CSDNN-WCE improve the recall. For the Texas Full dataset, the CSDNN-FL model has a recall of 61.6% versus 11.8% for the standard DNN model, which indicates the CSDNN-FL algorithm is capable of detecting more preeclamptic women than the standard DNN model.

**Table 14:** Comparison of CSDNN-FL and CSDNN-WCE versus DNN with and without Balanced Batches (BB) on the Full Texas, Oklahoma, and MOMI datasets as well as sub-population datasets. ACC, SP, PR, and RE represents accuracy, specificity, precision, and recall, respectively. The highest G-mean and AUC values are denoted in bold.

Dataset	Method	ACC	AUC	G-mean	RE	SP	PR
TX Full	CSDNN-FL	0.619	**0.663**	**0.617**	0.616	0.619	0.063
	CSDNN-WCE	0.813	0.663	0.590	0.420	0.830	0.093
	DNN	0.963	0.658	0.344	0.118	0.998	0.689
	CSDNN-FL-BB	0.831	0.634	0.572	0.385	0.850	0.096
	CSDNN-WCE-BB	0.040	0.633	0.000	1.000	0.000	0.040
	DNN-BB	0.832	0.634	0.571	0.384	0.851	0.096
TX AA	CSDNN-FL	0.748	**0.667**	**0.623**	0.512	0.762	0.110
	CSDNN-WCE	0.795	0.667	0.605	0.450	0.815	0.123
	DNN	0.951	0.665	0.414	0.173	0.996	0.689
	CSDNN-FL-BB	0.778	0.666	0.612	0.472	0.795	0.117
	CSDNN-WCE-BB	0.054	0.667	0.000	1.000	0.000	0.054
	DNN-BB	0.789	0.667	0.608	0.458	0.808	0.121
TX NA	CSDNN-FL	0.544	**0.571**	**0.535**	0.582	0.542	0.044
	CSDNN-WCE	0.658	0.563	0.484	0.413	0.666	0.043
	DNN	0.965	0.535	0.000	0.000	1.000	0.167
	CSDNN-FL-BB	0.502	0.500	0.285	0.498	0.502	0.047
	CSDNN-WCE-BB	0.426	0.492	0.282	0.584	0.420	0.045
	DNN-BB	0.706	0.571	0.466	0.368	0.718	0.046
OK Full	CSDNN-FL	0.622	**0.635**	**0.594**	0.566	0.626	0.082
	CSDNN-WCE	0.706	0.620	0.575	0.461	0.720	0.089
	DNN	0.944	0.620	0.000	0.000	1.000	0.000
	CSDNN-FL-BB	0.702	0.635	0.583	0.476	0.716	0.090
	CSDNN-WCE-BB	0.056	0.619	0.000	1.000	0.000	0.056
	DNN-BB	0.691	0.621	0.580	0.480	0.704	0.088
OK AA	CSDNN-FL	0.642	0.619	0.578	0.529	0.653	0.124
	CSDNN-WCE	0.589	**0.623**	**0.582**	0.588	0.589	0.115
	DNN	0.478	0.501	0.172	0.527	0.475	0.070
	CSDNN-FL-BB	0.710	0.594	0.479	0.374	0.740	0.128
	CSDNN-WCE-BB	0.082	0.554	0.000	1.000	0.000	0.082
	DNN-BB	0.582	0.581	0.551	0.533	0.586	0.105
OK NA	CSDNN-FL	0.701	**0.575**	**0.515**	0.386	0.724	0.091
	CSDNN-WCE	0.549	0.519	0.463	0.473	0.554	0.073
	DNN	0.8971	0.555	0.256	0.081	0.954	0.109
	CSDNN-FL-BB	0.592	0.528	0.473	0.443	0.602	0.076
	CSDNN-WCE-BB	0.501	0.502	0.460	0.498	0.502	0.066
	DNN-BB	0.708	**0.580**	**0.516**	0.386	0.730	0.094
MOMI	CSDNN-FL	0.694	0.756	0.682	0.672	0.696	0.176
	CSDNN-WCE	0.713	**0.765**	**0.690**	0.669	0.716	0.188
	DNN	0.904	0.735	0.433	0.195	0.971	0.396
	CSDNN-FL-BB	0.724	0.759	0.685	0.647	0.732	0.191
	CSDNN-WCE-BB	0.153	0.762	0.264	0.989	0.073	0.093
	DNN-BB	0.723	**0.768**	**0.690**	0.661	0.729	0.195
MOMI AA	CSDNN-FL	0.661	0.711	0.637	0.632	0.665	0.207
	CSDNN-WCE	0.722	**0.724**	**0.656**	0.591	0.739	0.234
	DNN	0.833	0.720	0.605	0.416	0.886	0.323
	CSDNN-FL-BB	0.757	**0.724**	**0.660**	0.559	0.783	0.251
	CSDNN-WCE-BB	0.539	0.703	0.263	0.754	0.511	0.168
	DNN-BB	0.752	0.694	0.631	0.512	0.783	0.235

**Fig 21:**
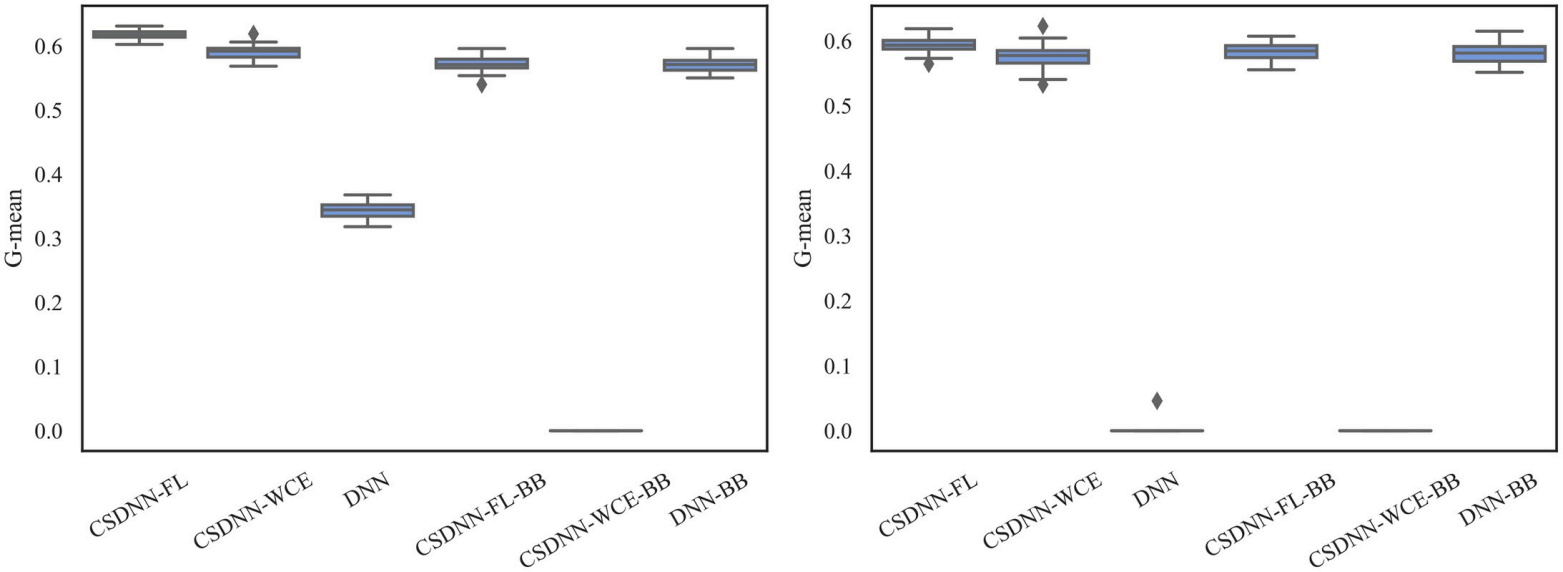
Comparison of CSDNN-FL and CSDNN-WCE versus DNN (in terms of G-mean) on the Full Texas and Oklahoma datasets. Each boxplot denotes variability of the G-mean (vertical axis) for different methods.

**Fig 22:**
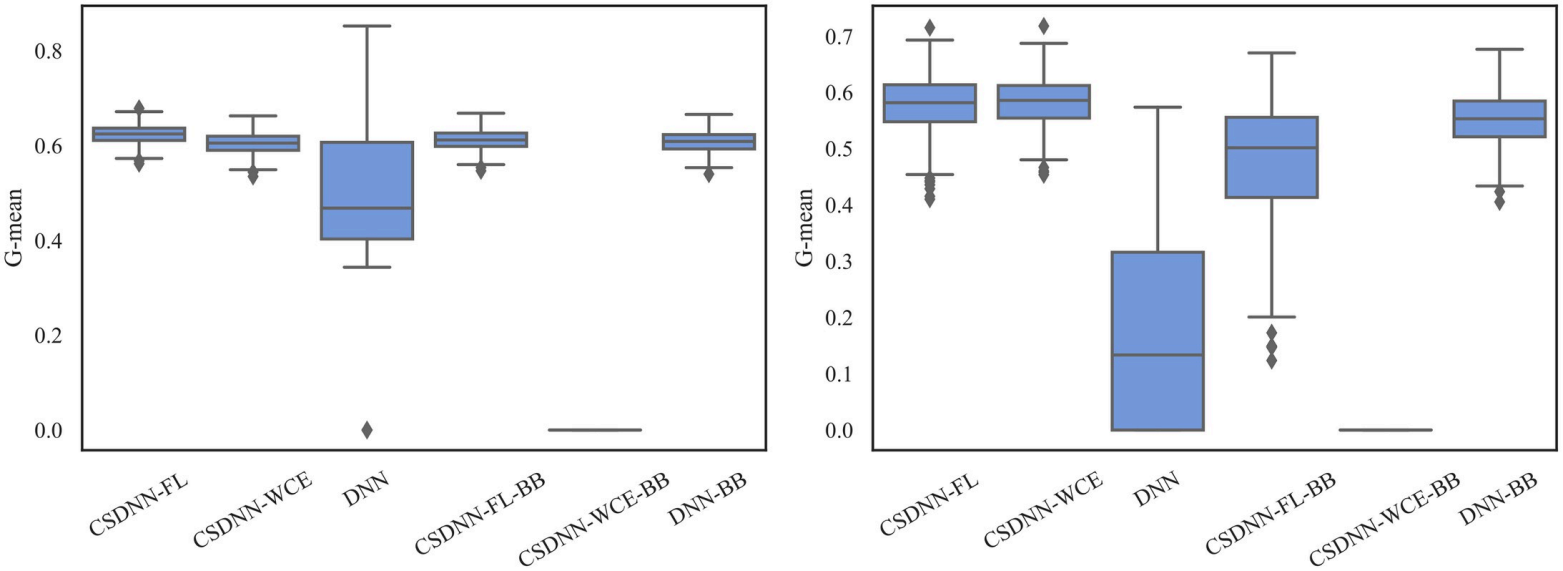
Comparison of CSDNN-FL and CSDNN-WCE versus DNN (in terms of G-mean) on the African American Texas and Oklahoma datasets. Each boxplot denotes variability of the G-mean (vertical axis) for different methods.

**Fig 23:**
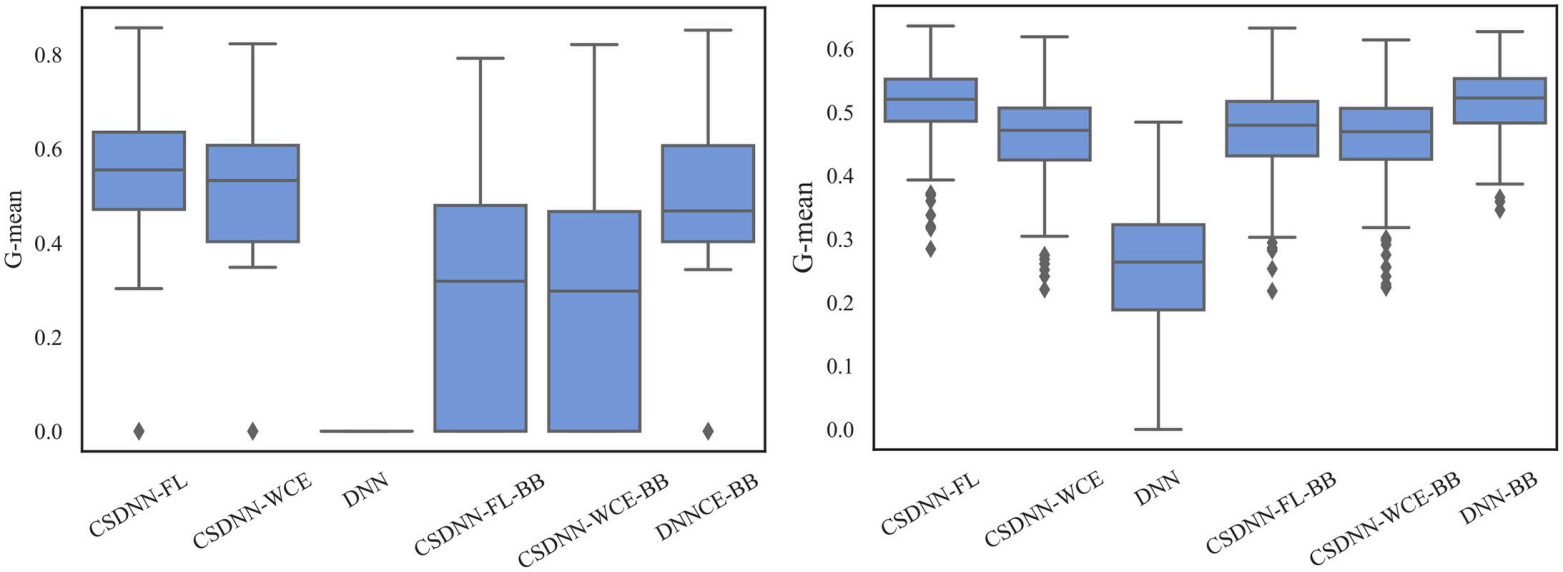
Comparison of CSDNN-FL and CSDNN-WCE versus DNN (in terms of G-mean) on the Native American Texas and Oklahoma datasets. Each boxplot denotes variability of the G-mean (vertical axis) for different methods.

**Fig 24:**
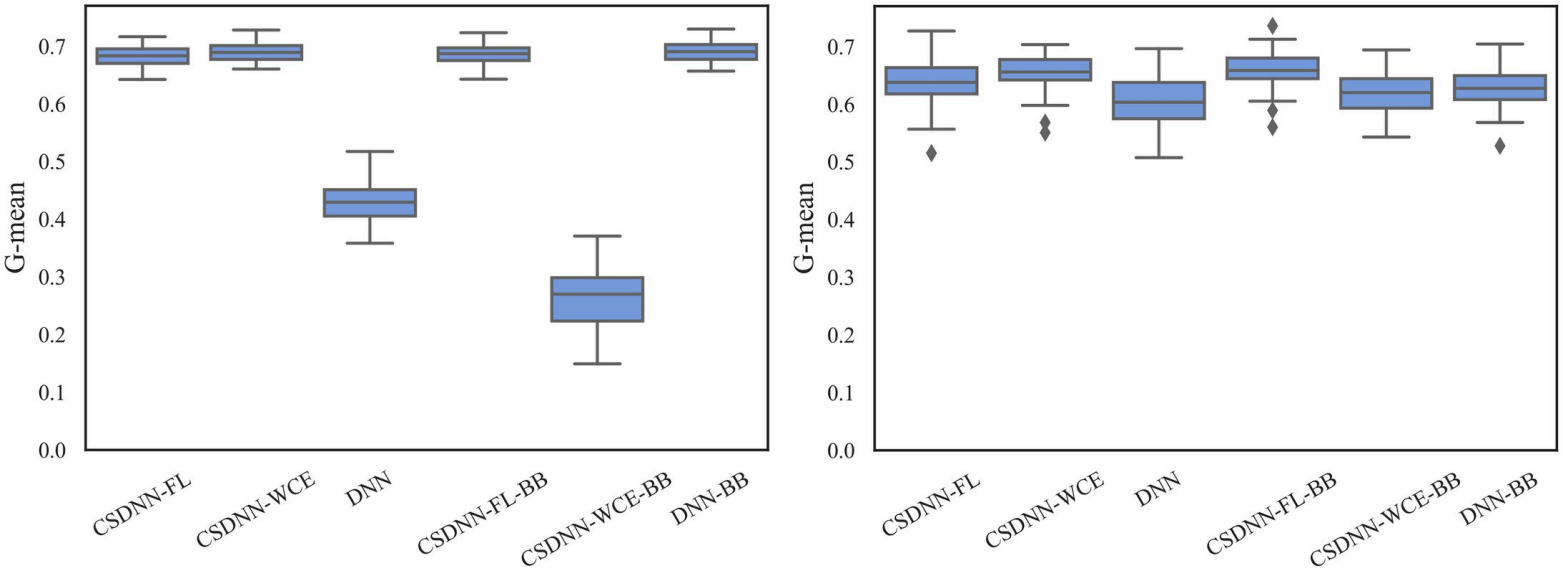
Comparison of CSDNN-FL and CSDNN-WCE versus DNN (in terms of G-mean) on the MOMI Full and African American populations. Each boxplot denotes variability of the G-mean (vertical axis) for different methods.

For measuring and comparing the characteristic of CSDNN-FL, CSDNN-WCE, and DNN with and without balanced batches, we used box plots for G-mean measure as shown in Figs 21–24, which have been obtained over 50 iterations on the same data for each algorithm for the Full datasets and 350 iterations on the same data for the subpopulations. As shown by the Figs 21–24 (and standard deviations in the S4 Table of the S1 File), CSDNN-FL was more robust than the other models in terms of G-mean. In addition, the models equipped with balanced batches more frequently demonstrated greater variation between runs for Oklahoma African American and Texas Native American datasets, which refleced their propensity for overfitting. Moreover, the African American subpopulation had more variation in G-mean in DNN compared to other methods for both Texas and Oklahoma datasets. We also observe that the Native American subpopulation amongst the Oklahoma dataset had a highly variable G-mean among others most likely due to the small datasets.

#### Comparative analysis of computation time

Table 15 shows the average time required for the proposed algorithms to complete training and testing. In addition to evaluating the quality of predictive models in terms of AUC and G-mean, it is critical to investigate whether there is a computational bottleneck. Each computational running time for training and testing is reported as an average of 10 iterations in which no hyperparameter tuning or cross validation was performed. Each model had the same architecture configuration with three hidden layers consisting of 60, 30, and 45 nodes in each layer respectively, and with a batch size of 8192. The MOMI data was an exception, in which each model was implemented with a batch size of 4096. We observed that the proposed algorithms were capable of dealing with large amounts of data in a reasonable amount of time.

**Table 15:** Average computational running time (in seconds) of each proposed model.

Model	TX Full	OK Full	MOMI Full
CSDNN-FL	46.573	15.068	37.870
CSDNN-WCE	45.336	14.827	37.849
DNN	49.934	15.332	37.260
CSDNN-FL-BB	75.240	20.203	39.858
CSDNN-WCE-BB	76.295	20.180	38.738
DNN-BB	80.896	19.613	39.762

#### Comparative analysis with traditional ml algorithms

To further evaluate the performance of the CSDNN methods, we compared our CSDNNs with multiple existing methods: logistic regression (LR), support vector machine with linear kernel (SVM-Lin), support vector machine with radial basis function (SVM-RBF), and cost-sensitive versions of each of those models including weighted LR (WLR), weighted SVM-Lin, (WSVM-Lin), and weighted SVM-RBF (WSVM-RBF). Tables 16–18 show the average G-mean and AUC values for the Texas, Oklahoma, and MOMI datasets, respectively. In all cases, the cost-sensitive versions outperform in terms of both AUC and G-mean, however the best performing model for the Texas and Oklahoma datasets is the CSDNN-FL with 66% and 64% AUC. The MOMI dataset demonstrated CSDNN-WCE and CSDNN-FL perform well compared to other techniques with 76% AUC. However, WLR produced the highest G-mean values followed by WSVM-RBF and CSDNNs methods.

**Table 16:** Comparative results of CSDNN-WCE and CSDNN-FL against Logistic Regression (LR), Weighted LR, Support Vector Machine (SVM-Lin), Weighted SVM-Lin (WSVM-Lin), SVM with Radial Basis Function (SVM-RBF), Weighted SVM-RBF (WSVM-RBF), and DNN using Texas data. The highest average G-mean and AUC values are denoted in bold.

	LR	WLR	SVM-Lin	WSVM-Lin	SVM-RBF	WSVM-RBF	DNN	CSDNN-WCE	CSDNN-FL
**G-mean**	0.013	0.579	0.000	0.523	0.329	0.607	0.344	0.590	**0.617**
**AUC**	0.500	0.596	0.500	0.605	0.553	0.621	0.661	0.663	**0.663**
**Recall**	0.117	0.500	0.000	0.300	0.108	0.489	0.118	0.420	0.616
**Specificity**	0.998	0.753	1.000	0.907	0.998	0.753	0.830	0.619	0.619
**Precision**	0.691	0.077	0.000	0.131	0.688	0.076	0.689	0.093	0.063

**Table 17:** Comparative results of CSDNN-WCE and CSDNN-FL against Logistic Regression (LR), Weighted LR, Support Vector Machine (SVM-Lin), Weighted SVM-Lin (WSVM-Lin), SVM with Radial Basis Function (SVM-RBF), Weighted SVM-RBF (WSVM-RBF), and DNN using Oklahoma data. The highest average G-mean and AUC values are denoted in bold.

	LR	WLR	SVM-Lin	WSVM-Lin	SVM-RBF	WSVM-RBF	DNN	CSDNN-WCE	CSDNN-FL
**G-mean**	0.012	0.576	0.000	0.515	0.000	0.561	0.001	0.575	**0.594**
**AUC**	0.500	0.596	0.500	0.579	0.500	0.582	0.620	0.620	**0.635**
**Recall**	0.001	0.456	0.000	0.300	0.000	0.419	0.000	0.461	0.566
**Specificity**	0.999	0.735	1.000	0.854	0.999	0.737	1.000	0.720	0.626
**Precision**	0.128	0.092	0.000	0.108	0.065	0.086	0.000	0.089	0.082

**Table 18:** Comparative results of CSDNN-WCE and CSDNN-FL against Logistic Regression (LR), Weighted LR, Support Vector Machine (SVM-Lin), Weighted SVM-Lin (WSVM-Lin), SVM with Radial Basis Function (SVM-RBF), Weighted SVM-RBF (WSVM-RBF), and DNN using MOMI data. The highest average G-mean and AUC values are denoted in bold.

	LR	WLR	SVM-Lin	WSVM-Lin	SVM-RBF	WSVM-RBF	DNN	CSDNN-WCE	CSDNN-FL
**G-mean**	0.306	**0.706**	0.000	0.677	0.118	0.699	0.433	0.690	0.682
**AUC**	0.544	0.708	0.500	0.691	0.507	0.703	0.735	**0.765**	0.756
**Recall**	0.094	0.654	0.000	0.610	0.007	0.626	0.195	0.669	0.672
**Specificity**	0.992	0.746	1.000	0.771	0.999	0.763	0.917	0.716	0.696
**Precision**	0.539	0.197	0	0.205	0.540	0.201	0.396	0.188	0.176

**Fig 25:**
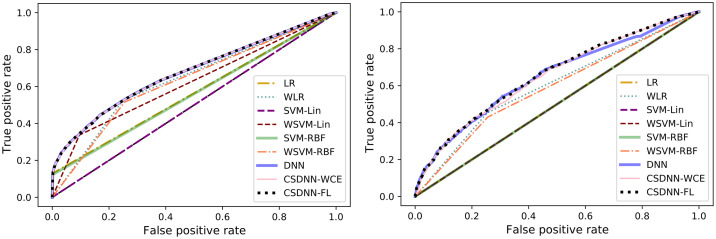
ROC curve for the Texas and Oklahoma dataset.

**Fig 26:**
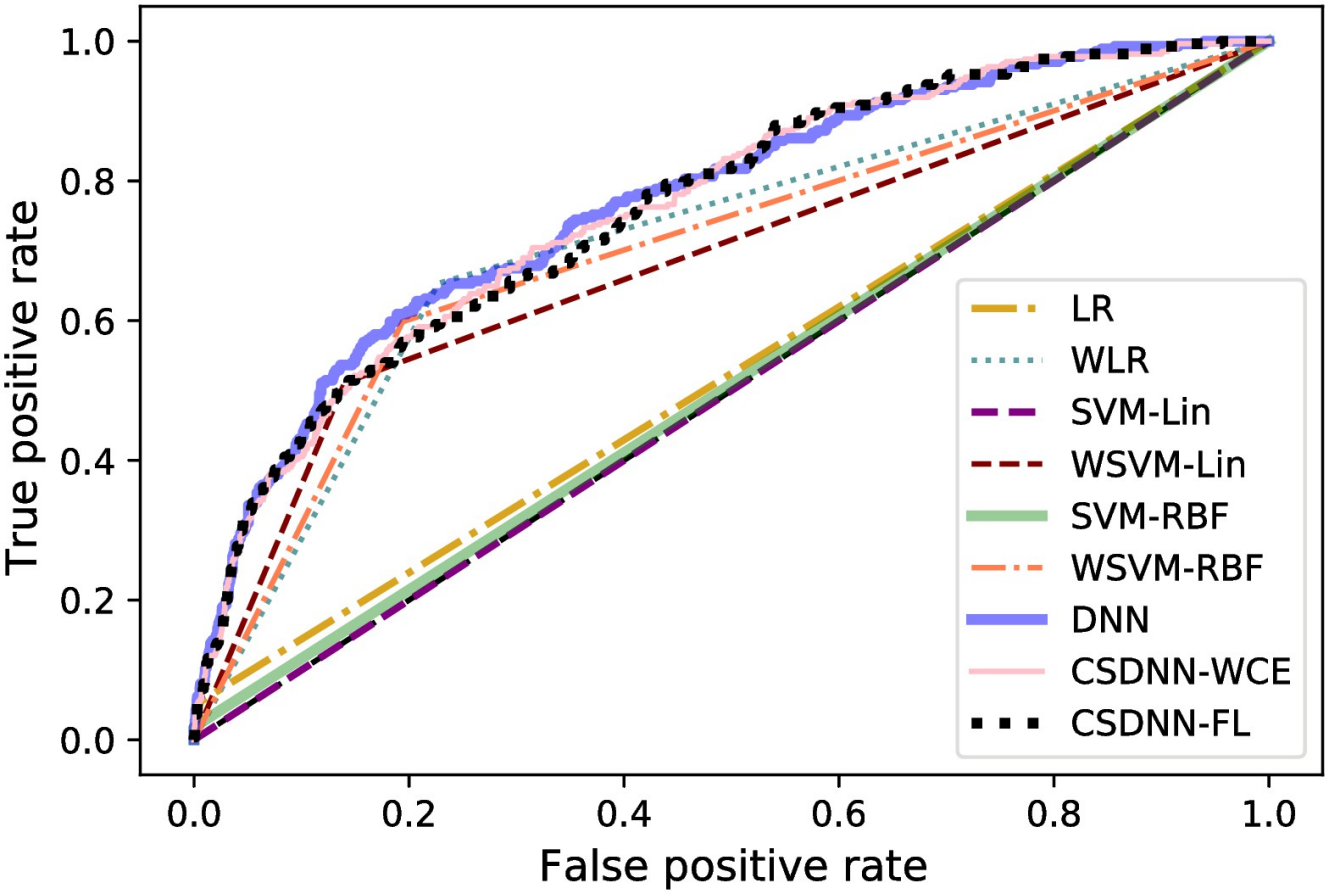
ROC curve for the MOMI dataset.

The ROC curve of all models is shown in Figs 25 and 26. These graphs show an improvement over other traditional algorithms, although in all datasets neural networks tend to perform similarly regardless of the loss function used (in terms of AUC), while the MOMI dataset’s CSDNN seemed to outperform DNN. The CSDNN models demonstrate significant superiority over LR and SVM for all datasets. Even though it is not easy to verify a specific winning technique from the curves, CSDNN-FL and CSDNN-WCE are the most promising prediction methods as demonstrated by the AUC shown in Tables 16–18. We note that the results of the pairwise Wilcoxon rank sum test are summarized in the S16–S18 Tables of the S1 File and are described in the Statistical Analysis of Results Section.

**Fig 27:**
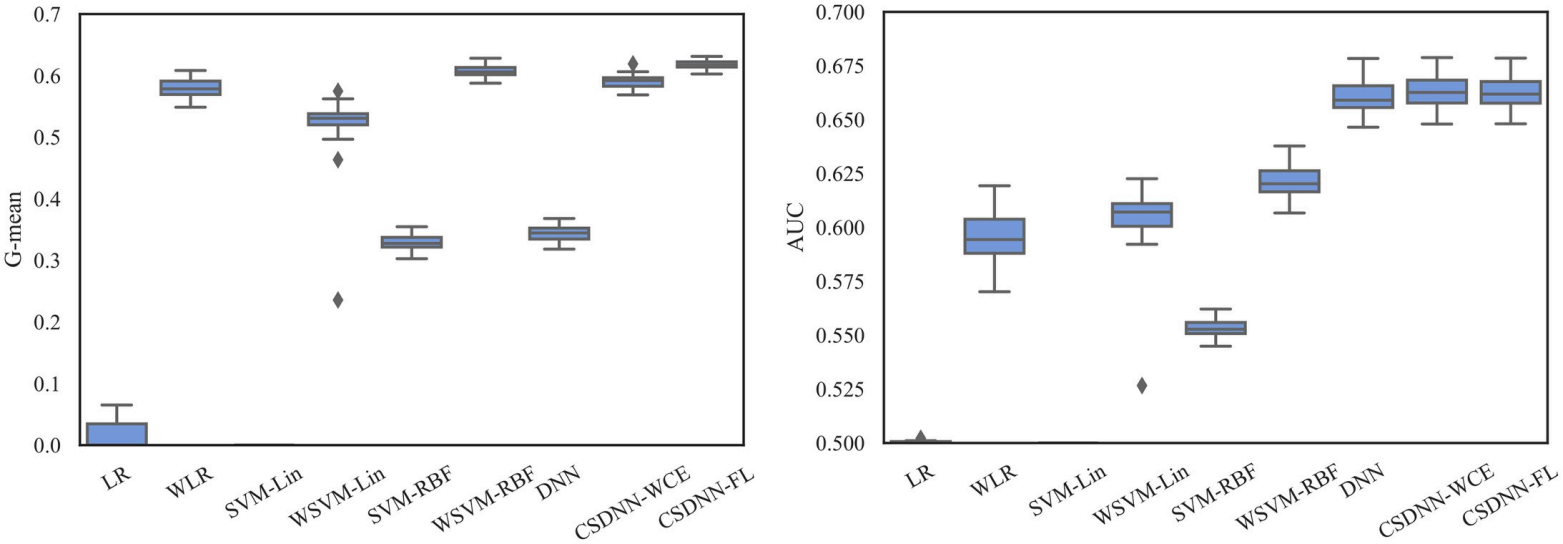
Robustness of CSDNN-FL and CSDNN-WCE in comparison to other ML algorithms for Texas dataset in terms of G-mean (left) and AUC (right).

**Fig 28:**
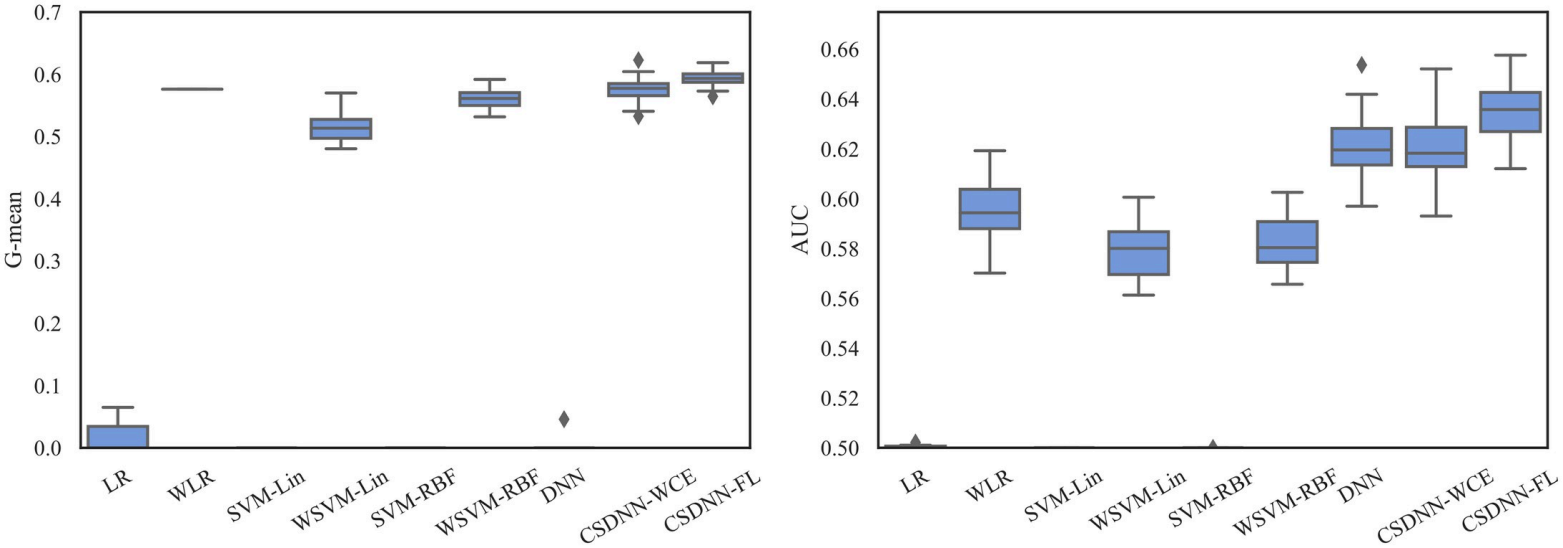
Robustness of CSDNN-FL and CSDNN-WCE in comparison to other ML algorithms for Oklahoma dataset in terms of G-mean (left) and AUC (right).

**Fig 29:**
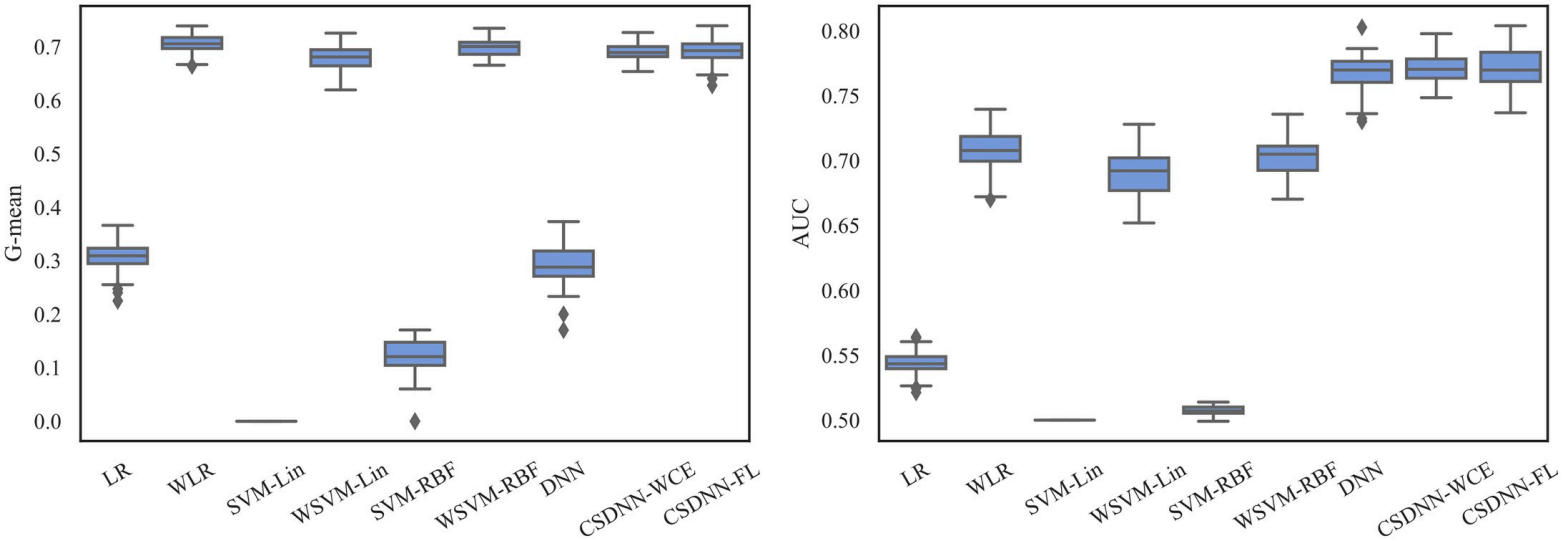
Robustness of CSDNN-FL and CSDNN-WCE in comparison to other ML algorithms for MOMI dataset in terms of G-mean (left) and AUC (right).

The robustness of the ML algorithm is critical in the PE prediction problem—a promising prediction method should produce the same results over several iterations. To measure this, we employed box plots as shown in Figs 27–29, which have been obtained over 50 iterations on the same data for each algorithm. As the figure shows, CSDNN-FL was more robust than the other algorithms for Oklahoma and Texas datasets. A small standard deviation was observed in CSDNN-FL followed by CSDNN-WCE for both datasets. WLR followed by CSDNN-WCE showed better results each time for the MOMI dataset, while the performance of both LR and SVM was inferior in most cases. Therefore, integrating cost-sensitive into prediction models could improve the accuracy of models.

#### Statistical analysis of results

To test whether there is a statistical difference between the models, a Kruskal-Wallis test was performed for each dataset. Table 19 shows that the null hypothesis is rejected with an extremely low p-value for each dataset (at a specific significance rate *α* = 0.05). We conclude that there is a statistical difference between the models.

**Table 19:** Kruskal-Wallis test for all three datasets.

Dataset	p-value	Hypothesis (*α* = 0.05)
**Texas**	≪ 0.05	Rejected *H*_0_
**Oklahoma**	≪ 0.05	Rejected *H*_0_
**MOMI**	≪ 0.05	Rejected *H*_0_

In order to test whether our CSDNN models (CSDNN-Focal and CSDNN-WCE) perform well compared to other existing methods, we perform a pairwise Wilcoxon rank sum test between the CSDNN models and the benchmark methods. This test is performed on G-mean values which are obtained from the 10-fold cross validation repeated 5 times. Since this test must be run multiple times, the family-wise error rate is taken into account by reducing the significance level to 0.0005.

The results of a one-tailed Wilcoxon rank-sum test between CSDNNs and the benchmark methods for the Texas, Oklahoma, and MOMI Full datasets are presented in the S16–18 Tables of the S1 File. The null hypothesis is rejected if the p-value for the test is lower than the significance rate *α* = 0.0005. CSDNN-FL and CSDNN-WCE significantly outperforms the corresponding methods in both Texas and Oklahoma datasets as shown in the S16 and 17 Tables of the S1 File. While CSDNN-WCE method outperformed most methods, it showed significantly inferior results compared to the WSVM-RBF in the Texas dataset, and the WLR, CSDNN-FL-BB, and DNN-BB in the Oklahoma dataset. However, our CSDNN models performed significantly better than most methods, except WLR, WSVM-RBF, DNN-BB, and CSDNN-FL-BB for the MOMI dataset (S18 Table of the S1 File).

The results of the one-tailed Wilcoxon rank test for Texas and Oklahoma African American datasets are shown in S19 and S20 Tables in the S1 File, respectively. For these datasets, CSDNN-FL significantly outperformed the other methods except for the Oklahoma African American dataset, in which CSDNN-WCE performed significantly better than the CSDNN-FL.

The one-tailed Wilcoxon rank-sum test results for Texas and Oklahoma Native American datasets are presented in S21 and S22 Tables in the S1 File. We observed that CSDNN-FL significantly outperformed most methods, except DNN-BB, in both datasets, however, there was no significant difference between CSDNN-FL and CSDNN-WCE in Oklahoma Native American dataset.

CSDNN-FL and CSDNN-WCE performed significantly better than DNN and CSDNN-WCE-BB for MOMI African American dataset, while there is no significant difference between CSDNN-FL, CSDNN-WCE, CSDNN-FL-BB, and DNN-BB as shown in the S23 Table in the S1 File.

## Conclusion

False-negative PE predictions may result in high rates of maternal morbidity and mortality, while false positives may lead to unnecessary interventions. As such, identifying patients who would be well suited for outpatient management is challenging. Providing physicians with reliable and accurate tools to improve targeting and implementation prevention measures is critical in advancing the life-long health of preeclamptic patients. We propose the use of CSDNN in PE prediction, which suffers from highly imbalanced datasets. We compared the focal loss function in CSDNN (originally applied to image data) with both weighted cross-entropy and standard cross-entropy loss functions. In addition, we evaluated and compared the results of CSDNNs with the corresponding models equipped with balanced batch training sets obtained from random oversampling.

We performed an extensive experimental analysis using three clinical datasets to show the advantages of our CSDNN algorithm. Provided that the African American and Native American women experience severe morbidity and mortality rates compared to of their Caucasian counterparts during pregnancy, we studied the performance of our method on each sub-population in addition to the full datasets. We further compared the CSDNN results with the performance of the existing methods. Our results demonstrated that in many cases (5 out of 8 datasets), our CSDNN equipped with focal loss function performed better with significantly less variation in the results compared to other methods in terms of G-mean and AUC.

Limitations to this study largely involve Oklahoma and Texas PUDFs which do not include laboratory test results, detailed drug or alcohol usage, detailed blood pressure, specific height/weight information, etc. To overcome this drawback, we studied MOMI data which contains granular information about prenatal visits. Furthermore, Texas PUDF do not allows us to distinguish multiple-incident events for the same patient, which is necessary to treat the bias in our statistical modelling results. To overcome this issue, we studied the Texas PUDF data for only one year.

Future studies should extend the application of models to early and late onset PE. In addition, while our model accounts for race/ethnicity in PE prediction and presents promising classification results for each group in a highly imbalanced setting, additional investigation and computational testing for more equitable results, and further data collection for the minority groups needs to be explored in the future. The proposed models for minority groups can be extended to other health problems with disparities in outcomes. We will improve the results of the deep neural network on specially small datasets in the future works [93]. In addition, future studies should include detailed information on socioeconomic status, maternal weathering, and allostatic load. Fairness in machine learning also merits continued investigation.

## Supporting information

S1 File File of supporting results and data analysis. (ZIP)
