# Supplementary material for: An imbalance-aware deep neural network for early prediction of preeclampsia
Source: PLoS One. 2022 Apr 6;17(4):e0266042. doi: 10.1371/journal.pone.0266042 (PMC8985991; doi:10.1371/journal.pone.0266042)
Supplement: S1 File — (ZIP) [file pone.0266042.s001.zip › S1.pdf]

Supplementary Materials

In this section, the acronyms are described in S1 Table-S2 Table. The ICD-9-CM and ICD-10-CM codes for the clinical attributes and outcomes are listed in S3 Table. The standard deviation of the methods shown in Figs 20-23 are demonstrated in S4 Table.

S1 Table. Table of acronyms

Acronym	Term
ICD-CM	International Classification of Disease - Clinical Modification
B	Batch size
T	Number of epochs
h	Number of hidden layers in neural network
k	Number of nodes per layer
a	Activation function
η	Learning rate of neural network
TH	Hyperbolic tangent (*tanh*) activation function
RL	Rectified Linear Unit (*ReLU*) activation function
RS	Random search
HB	Hyperband
BO	Bayesian optimization
DNN	Deep neural network
CSDNN	Cost sensitive deep neural network
WCE	Weighted cross entropy
FL	Focal loss
ACC	Accuracy
SP	Specificity
PR	Precision
RE	Recall
BB	Balanced batch
LR	Logistic regression
SVM-Lin	Support vector machine with linear kernel
SVM-RBF	Support vector machine with radial basis function
WLR	Weighted logistic regression
WSVM-Lin	Weighted support vector machine with linear kernel
WSVM-RBF	Weighted support vector machine with radial basis function

S3 Table. ICD-9-CM and ICD-10-CM codes used in this study. Each diagnosis variable is searched for codes beginning with the below characters.

Disease	ICD-9-CM	ICD-10-CM
Obesity	V853, V854, 27800, 27801, 27803, 6491	E66, O9921, O9981, O9984, Z683, Z684, Z713, Z9884
Pregnancy resulting from assisted reproductive technology	V2385	O0981
Cocaine dependence	3042, 3056	F14, T405
Amphetamine dependence	3044, 3057	F15, F19, P044, T4362
Gestational diabetes mellitus	6488	O244, P700

Continued on next page

S3 Table – *Continued from previous page*

Pre-existing diabetes mellitus	250, 6480	E10, E11, O240, O241, O243, O248, O249
Anxiety	3000	F064, F41
Anemia NOS	2859	D51
Iron deficiency anemia	280	D50
Other anemia	281	D64, D59, D489, D53, O990
Depression	311	F32, F341, F33, F0631, Z139, Z1331, Z1332
Primigravidas at the extremes of maternal age	6595, V2381, V2383	O095, O096
Hemorrhagic disorders due to intrinsic circulating antibodies	2865	D683
Systemic lupus erythematosus	7100	M32
Lupus erythematosus	6954	L93, D6862
Autoimmune disease not elsewhere classified	27949	D89
Pure hypercholesterolemia	2720	E780
Unspecified vitamin D deficiency	2689	E55
Proteinuria	7910	D511, N06, O121, O122, R80
Tobacco use disorder	3051, 6490	-
History of tobacco use	V1582	-
Current Smoker	-	F172
Hypertension	401	G932, I10, I14, I15, I272, I674, I973, O10, O13, O16, R030
Hypertensive heart disease	402	I11
Chronic venous hypertension	4593	I873
Unspecified renal disease in pregnancy without mention of hypertension	6462	O2683, O9089
Chronic kidney disease	585	D631, E0822, E0922, E0922, E1022, E1122, E1322, N18
Hypertensive kidney disease	403	I12
Hypertensive heart and chronic kidney disease	404	I13
Renal failure not elsewhere classified	586	N19
Infections of genitourinary tract in pregnancy	6466	O23, O861, O862, O868
UTI	5990	O0338, O0388, O0488, O0788, O0883, N136, N390, N99521, N99531
Personal history of trophoblastic disease	V131	Z8759, O01
Supervision of high-risk pregnancy with history of trophoblastic disease	V231	O091
Thrombophilia	28981	D685, D686
History of premature delivery	V1321	Z8751
Hemorrhage in early pregnancy	640	O20

Continued on next page

S3 Table – *Continued from previous page*

Congenital abnormalities of the uterus including those complicating pregnancy, childbirth, or the puerperium	6540, 7522, 7523	O34, O340
Multiple Gestations	651	O30
Fetal Growth Restriction	764	O093
Asthma	493	
Obstructive Sleep Apnea	32723	
Other cardiovascular diseases complicating pregnancy and childbirth or the puerperium	6486	O9943
Sickle cell disease	28260	D57
Thyroid Disease	240, 241, 242, 243, 244, 245, 246	E00, E01, E02, E03, E04, E05, E06, E07
Inadequate Prenatal Care	V237	O093
Periodontal disease	523	E08630, E09630, E10630, E11630, E13630, K05, K06, K08129
Preeclampsia/Eclampsia	6424, 6425, 6426, 6427	O14, O15

S4 Table. The standard deviation (SD) of methods related to Figs 20-23

Dataset	Method	SD
TX Full	CSDNN-FL	0.006
	CSDNN-WCE	0.010
	DNN	0.012
	CSDNN-FL-BB	0.011
	CSDNN-WCE-BB	0.000
	DNN-BB	0.010
TX AA	CSDNN-FL	0.020
	CSDNN-WCE	0.022
	DNN	0.213
	CSDNN-FL-BB	0.021
	CSDNN-WCE-BB	0.000
	DNN-BB	0.022
TX NA	CSDNN-FL	0.149
	CSDNN-WCE	0.195
	DNN	0.000
	CSDNN-FL-BB	0.233
	CSDNN-WCE-BB	0.226
	DNN-BB	0.213
OK Full	CSDNN-FL	0.011
	CSDNN-WCE	0.017
	DNN	0.009
	CSDNN-FL-BB	0.013
	CSDNN-WCE-BB	0.000
	DNN-BB	0.015

Continued on next page

S4 Table – *Continued from previous page*

OK AA	CSDNN-FL	0.052
	CSDNN-WCE	0.045
	DNN	0.175
	CSDNN-FL-BB	0.106
	CSDNN-WCE-BB	0.000
	DNN-BB	0.050
OK NA	CSDNN-FL	0.054
	CSDNN-WCE	0.067
	DNN	0.101
	CSDNN-FL-BB	0.067
	CSDNN-WCE-BB	0.067
	DNN-BB	0.053
MOMI	CSDNN-FL	0.016
	CSDNN-WCE	0.018
	DNN	0.048
	CSDNN-FL-BB	0.019
	CSDNN-WCE-BB	0.050
	DNN-BB	0.022
MOMI AA	CSDNN-FL	0.032
	CSDNN-WCE	0.037
	DNN	0.086
	CSDNN-FL-BB	0.026
	CSDNN-WCE-BB	0.048
	DNN-BB	0.028

S5 Table shows the statistics of common comorbidities of the entire cohort and the two individual study groups. These comorbidities include the urinary tract infection (UTI), unspecified renal disease in pregnancy without mention of hypertension (renal disease), supervision of pregnancy, supervision of high-risk pregnancy with history of trophoblastic disease (TRO), congenital abnormalities of the uterus including those complicating pregnancy (Congenital abnormalities), other cardiovascular disease complicating pregnancy and childbirth, or the puerperium (Other cardiovascular), chronic kidney disease (CKD), autoimmune disease not elsewhere classified, primigravida at the extremes of maternal age, hemorrhagic disorders due to intrinsic circulating antibodies, renal failure not elsewhere classified, infections of genitourinary (GU) tract in pregnancy, personal history of trophoblastic disease, congenital abnormalities of the uterus, and GU. The vast majority of patients do not have many of the clinical diagnoses which leads to an extremely sparse dataset.

S6 Table. Clinical features of the MOMI dataset

**Feature**	**Frequency**
Acute Renal Failure	97 (0.309%)
Asthma	3,485 (11.088%)
Autoimmune Diseases	3 (0.010%)
Bacterial Vaginosis	20 (0.064%)
CKD	56 (0.178%)
CNS Abnormality	
Spina Bifida	3 (0.010%)
Congenital Hydrocephalus	7 (0.022%)
Multiple Diagnostic Codes	12 (0.038%)

Continued on next page

S6 Table – *Continued from previous page*

Microcephaly	178 (0.566%)
Other Congenital Illness	30,785 (97.945%)
Chlamydia	1,531 (4.871%)
Chronic Hypertension	2,010 (6.395%)
Cocaine	90 (0.286%)
Condylomata	64 (0.204%)
Congenital Syphilis	0 (98.947%)
Depression	5,174 (83.539%)
Diabetes Mellitus	
Unspecified Prior Diabetes	24 (0.639%)
Type I	201 (0.970%)
Type II	305 (7.359%)
Gestational Diabetes	2,313 (90.955%)
None	28,588 (90.955%)
Gestational Hypertension	2,875 (9.147%)
Gonococcal Infection	9 (0.029%)
Group B Streptococcus	7,952 (25.300%)
Heart Failure	0 (0.000%)
Hemorrhagic Disorder	1 (0.003%)
Hepatitis B Infection	47 (0.150%)
Maternal Herpes Infection or History of Herpes	1,676 (5.332%)
Personal History of Trophoblastic Disease	25 (0.080%)
History of Infertility	11 (0.035%)
History of Premature Delivery	14 (0.045%)
High-risk Pregnancy with History of Trophoblastic Disease	2 (0.006%)
Hyperemesis Gravidarum	22 (0.070%)
Periodontal Disease	2 (0.006%)
Previous Cesarean	4,263 (13.563%)
Primigravida	13,804 (43.918%)
Proteinuria	121 (0.385%)
Repeat Cesarean	4,689 (14.918%)
Sickle Cell Anemia with Crisis	4 (0.013%)
Internal Injuries of Thorax, Abdomen, and Pelvis	1 (0.003%)
Thrombocytopenia	
Other	12 (0.038%)
Disseminated Intravascular Coagulation	14 (0.045%)
Gestational	498 (1.584%)
None	30,122 (95.835%)
Thrombophilia	284 (0.904%)
Kidney Disease	
Lupus Nephritis	1 (0.003%)
Pyelonephritis	2 (0.006%)
Glomerulonephritis	5 (0.016%)
Transplant	5 (0.016%)
Nephrotic Syndrome	26 (0.083%)
Nephrolithiasis	51 (0.162%)
Multiple Diagnostic Codes	57 (0.181%)
Other	83 (0.264%)
None	31,196 (99.252%)
Anemia without Hemoglobinopathy	
Folate Deficiency Anemia	3 (0.01%)

Continued on next page

S6 Table – *Continued from previous page*

Unspecified Anemia	11 (0.035%)
B2 Deficiency Anemia	89 (0.283%)
Iron Deficiency Anemia	763 (2.428%)
None	30,565 (97.245%)
Collagen Vascular Disease	
Multiple Diagnostic Codes	1 (0.003%)
Rheumatoid Arthritis	68 (0.216%)
Lupus	87 (0.277%)
None	31,275 (99.504%)
Hemoglobinopathy	
Hemoglobin (Hgb)-SC	3 (0.01%)
Alpha Thalassemia	4 (0.013%)
Beta Thalassemia	7 (0.022%)
Hgb-SS	10 (0.032%)
Hgb-Sthal	66 (0.21%)
Sickle Cell Trait	390 (1.241%)
None	30,951 (98.473%)
Maternal Liver, Gall Bladder, or Pancreatic Illness	
Hepatitis A	1 (0.003%)
LiverTransplant	6 (0.019%)
Pancreatitis	7 (0.022%)
Other	27 (0.086%)
Hepathesis B	41 (0.13%)
Cholelithiasis	427 (1.359%)
Hepatitis C	436 (1.387%)
None	30,486 (96.993%)
Structural Heart Disease	
Artificial Valves	3 (0.01%)
Myocarditis/Cardiomyopathy	19 (0.06%)
Rheumatic Heart Disease	45 (0.143%)
Other	64 (0.204%)
Valve Disorder	92 (0.293%)
Congenital Heart Disease	93 (0.296%)
None	31,115 (98.995%)
Marijuana Use	967 (3.077%)
Maternal Neuromuscular Disease	
Cerebral Palsy	3 (0.010%)
Myotonic Dystrophy	4 (0.013%)
Myasthenia Gravis	9 (0.029%)
Multiple Sclerosis	55 (0.175%)
None	31,346 (99.730%)
Operations On Heart and Pericardium	5 (0.016%)
Opioid Abuse	651 (2.071%)
Other Substance Abuse	
Hallucinogens	1 (0.003%)
Sedatives/Hypnotics/Anxiolytics	1 (0.003%)
Stimulants	2 (0.006%)
Anti-Depressants/Other Psychoactive	10 (0.032%)
Alcohol	17 (0.054%)
Multiple Diagnostic Codes	38 (0.121%)
Other	2,487 (7.913%)

Continued on next page

S6 Table – *Continued from previous page*

None	28,875 (91.868%)
Total Number of Pregnancies	
Avg	2.410
SD	1.625
Min	1
Max	31
Deliveries Prior to Admission	
Avg	0.919
SD	1.110
Min	0
Max	13
Total Abortions	
Avg	0.491
SD	0.940
Min	0
Max	20
Mean Arterial Pressure (MAP)	
Avg	84.261
SD	8.162
Min	38
Max	140.667
Previous Incidents of High Blood Pressure	
Avg	0.342
SD	0.623
Min	0
Max	5

Missing Data

S7 Table, S8 Table, and S9 Table show the list of features with missing values in the Texas PUDF, Oklahoma, and MOMI datasets, respectively.

Model Selection

The best architecture along with hyperparameters obtained from the three model selection techniques for the best architecture of the DNN and CSDNN with WCE and FL functions as well as hybrid models that further balances batches with oversampling (Balanced Batches) are summarized in Tables 10-15. In these tables, *tanh* is abbreviated as TH, *ReLU* is abbreviated as RL, Random Search is represented by RS, Hyperband is denoted by HB, and Bayesian optimization is represented by BO.

S2 Table. Table of acronyms

Acronym	Term
PE	Preeclampsia
ML	Machine Learning
FFS	Forward feature selection
BFS	Backward feature selection
M	Mean Imputation
EM	Expectation maximization
OS	Oversampling
SLM	Supervised Learning Mdoel
EN	Elastic net
DT	Decision Tree
RF	Random Forest
LR	Logistic Regression
SVM-Lin	Support vector machine with a linear kernel
SVM-RBF	Support vector machine with a radial basis function
WLR	Weighted logistic regression
WSVM-Lin	Weighted support vector machine with a linear kernel
WSVM-RBF	Weighted support vector machine with a radial basis function
ANN	Artificial Neural Network
GB	Gradient Boosting
EL	Ensemble Learning
NB	Naïve Bayes
GM	Gaussian Model
DNN	Deep neural network
CSDNN	Cost sensitive deep neural network
ACC	Accuracy
SP	Specificity
PR	Precision
RE	Recall
GM	G-Mean
FM	F-Measure
B	Batch size
T	Number of epochs
h	Number of hidden layers in neural network
k	Number of nodes per layer
a	activation function
η	Learning rate of neural network
TH	Hyperbolic tangent (*tanh*) activation function
RL	Rectified Linear Unit (*ReLU*) activation function
RS	Random Search optimization
HB	Hyperband Optimization
BO	Bayesian Optimization
WCE	Weighted Cross-entropy
FL	Focal Loss
BB	Balanced Batch

S5 Table. Patient clinical characteristics in the Texas and Oklahoma PUDF sets

Feature	Frequency	
	Texas	Oklahoma
Obesity	19,208 (5.32%)	7,136 (8.43%)
Pregnancy from assisted reproductive technology	615 (0.17%)	32 (0.04%)
Cocaine Dependence	0	67 (0.08%)
Amphetamine Dependence	0	962 (1.14%)
Gestational Diabetes Mellitus	21,658 (6.00%)	5,025 (5.94%)
Pre-existing Diabetes Mellitus	4,065 (1.13%)	1,159 (1.37%)
Anxiety	2,709 (0.75%)	3,148 (3.72%)
Anemia NOS	29,280 (8.11%)	11 (0.01%)
Iron Deficiency Anemia	3,937 (1.09%)	1,246 (1.47%)
Other Anemia	94 (0.03%)	12,784 (15.11%)
Depression	3,157 (0.88%)	2,752 (3.25%)
Primigravida	4,969 (1.38%)	1,796 (2.12%)
Hemorrhagic	6 (0.002%)	0
Systemic Lupus Erythematosus	366 (0.10%)	141 (0.17%)
Lupus Erythematosus	20 (0.006%)	35 (0.04%)
Autoimmune	18 (0.005%)	9 (0.01%)
Pure Hypercholesterolemia	108 (0.03%)	25 (0.03%)
Unspecified Vitamin D Deficiency	227 (0.06%)	189 (0.22%)
Proteinuria	21 (0.006%)	166 (0.20%)
Tobacco Use Disorder	6,140 (1.70%)	-
History of Tobacco Use	3,226 (0.89%)	-
Current Smoker	-	5,438 (6.43%)
Hypertension	2,424 (0.67%)	10,276 (12.14%)
Hypertensive Heart Disease	16 (0.004%)	5 (0.006%)
Chronic Venous Hypertension	1 (0.0003%)	1 (0.001%)
Unspecified Renal Disease	644 (0.18%)	546 (0.65%)
Chronic Kidney Disease	173 (0.05%)	73 (0.09%)
Hypertensive Kidney Disease	96 (0.03%)	24 (0.03%)
Hypertensive Heart and CKD	6 (0.002%)	2 (0.002%)
Renal Failure	6 (0.002%)	0
GU Tract Infection	3,299 (0.91%)	618 (0.73%)
UTI	1,838 (0.51%)	175 (0.21%)
History of Trophoblastic Disease	0	390 (0.46%)
Trophoblastic Disease	28 (0.008%)	11 (0.013%)
Thrombophilia	1073 (0.30%)	271 (0.32%)
History of Premature Delivery	180 (0.05%)	149 (0.18%)
Hemorrhage in Early Pregnancy	216 (0.06%)	22 (0.03%)
Uterus's Congenital Abnormalities	1,184 (0.33%)	17,082 (20.18%)
Multiple Gestations	5,871 (1.63%)	1,393 (1.65%)
Fetal Growth Restriction	3 (0.001%)	1 (0.001%)
Asthma	7,124 (1.98%)	3,547 (4.19%)
Obstructive Sleep Apnea	106 (0.03%)	58 (0.07%)
Other Cardiovascular Diseases	1,372 (0.38%)	46 (0.05%)
Sickle Cell Disease	75 (0.02%)	284 (0.34%)
Thyroid Disease	8,880 (2.46%)	2,750 (3.25%)
Inadequate Prenatal Care	8,959 (2.48%)	767 (0.91%)
Periodontal Disease	35 (0.01%)	2 (0.002%)

S7 Table. The list of features with missing values in the Texas dataset

Feature	Missing Ratio
Race	878 (0.24%)
Ethnicity	3,418 (0.95%)
County	9,018 (2.50 %)
Insurance	149 (0.04%)

S8 Table. The list of features with missing values in the Oklahoma PUDF

Feature	Missing Ratio
Marital Status	15,015 (17.742%)
Insurance	3 (0.004%)
County	3 (0.004%)

S9 Table. The list of features with missing values in the MOMI dataset

Feature	Missing Ratio
Insurance	7 (0.022%)
Number of Pregnancies	118 (0.375%)
Number of Deliveries Prior to Admission	118 (0.375%)
Number of Abortions	118 (0.375%)
Primigravida	118 (0.375%)
Weight At Admission	1513 (4.814%)
Infant Sex	416 (1.324%)
CNS Congenital Abnormality	331 (1.053%)
Congenital Syphilis	331 (1.053%)
UTI	331 (1.053%)
Race	647 (2.058%)
Prenatal Visit Weight	388 (1.234%)
MAP	439 (1.397%)

S10 Table. DNN architecture for Texas PUDF, Oklahoma PUDF and MOMI datasets. We note that h_i and a_i refer to the number of neurons and activation function in the hidden layer i, respectively, where $i = 1, 2, 3, ..., 8$.

**Dataset**	h_1, a_1	h_2, a_2	h_3, a_3	h_4, a_4	h_5, a_5	h_6, a_6	h_7, a_7	h_8, a_8	Optimizer	η	B	Tuner
**TX Full**	30, RL	30, TH	60, TH	60, RL	60, TH	45, RL	60, TH	30, RL	NAdam	0.001	8192	HB
**TX AA**	60, TH	30, RL	45, RL	-	-	-	-	-	RMSProp	0.001	8192	HB
**TX NA**	60, TH	30, RL	45, RL	-	-	-	-	-	RMSProp	0.001	8192	HB
**OK Full**	60, TH	60, RL	41, TH	-	-	-	-	-	RMSProp	0.001	8192	HB
**OK AA**	30, TH	60, RL	45, TH	45, RL	41, TH	-	-	-	SGD	0.0001	8192	HB
**OK NA**	36, TH	30, TH	60, TH	41, TH	36, TH	-	-	-	RMSprop	0.0001	8192	RS
**MOMI**	60, RL	30, TH	-	-	-	-	-	-	NAdam	0.001	8192	BO
**MOMI AA**	41, TH	41, TH	60, TH	-	-	-	-	-	RMSprop	0.001	8192	HB

S11 Table. CSDNN-WCE architecture for Texas PUDF, Oklahoma PUDF and MOMI datasets. We note that h_i and a_i refer to the number of neurons and activation function in the hidden layer i, respectively, where $i = 1, 2, 3, ..., 8$.

**Dataset**	h_1, a_1	h_2, a_2	h_3, a_3	h_4, a_4	h_5, a_5	h_6, a_6	h_7, a_7	h_8, a_8	Optimizer	η	B	Tuner
**TX Full**	45, TH	30, RL	60, TH	30, TH	30, TH	60, TH	30, TH	-	Adam	0.001	8192	HB
**TX AA**	36, RL	30, RL	45, RL	-	-	-	-	-	RMSprop	0.001	8192	HB
**TX NA**	41, RL	30, RL	36, TH	45, TH	-	-	-	-	RMSprop	0.001	8192	RS
**OK Full**	30, TH	60, TH	41, TH	-	-	-	-	-	RMSprop	0.001	8192	HB
**OK AA**	60, RL	60, TH	60, TH	30, TH	-	-	-	-	NAdam	0.001	8192	RS
**OK NA**	36, TH	41, TH	36, RL	30, RL	-	-	-	-	Adam	0.0001	8192	BO
**MOMI**	60, TH	30, TH	-	-	-	-	-	-	Adam	0.001	8192	BO
**MOMI AA**	30, TH	41, RL	-	-	-	-	-	-	RMSprop	0.001	8192	BO

S12 Table. CSDNN-FL architecture for Texas PUDF, Oklahoma PUDF and MOMI datasets. We note that h_i and a_i refer to the number of neurons and activation function in the hidden layer i, respectively, where $i = 1, 2, 3, ..., 8$.

**Dataset**	h_1, a_1	h_2, a_2	h_3, a_3	h_4, a_4	h_5, a_5	h_6, a_6	h_7, a_7	h_8, a_8	Optimizer	η	B	Tuner	α	γ
**TX Full**	60, TH	30, RL	45, RL	-	-	-	-	-	RMSProp	0.001	8192	HB	0.97	1.25
**TX AA**	60, TH	30, RL	45, RL	-	-	-	-	-	RMSProp	0.001	8192	HB	0.96	1.75
**TX NA**	60, TH	30, RL	45, RL	-	-	-	-	-	NAdam	0.001	8192	HB	0.97	1
**OK Full**	60, TH	30, RL	45, RL	-	-	-	-	-	RMSProp	0.001	8192	HB	0.95	1.0
**OK AA**	60, TH	30, RL	45, RL	-	-	-	-	-	RMSProp	0.001	8192	HB	0.92	0.25
**OK NA**	30, TH	30, TH	41, RL	60, RL	-	-	-	-	Adam	0.001	8192	HB	0.94	0.25
**MOMI**	45, RL	60, TH	41, TH	-	-	-	-	-	RMSprop	0.001	8192	BO	0.92	0.75
**MOMI AA**	30, TH	41, RL	-	-	-	-	-	-	RMSprop	0.001	8192	BO	0.90	1

S13 Table. CSDNN-FL (with Balanced Batches) architecture for Texas PUDF, Oklahoma PUDF and MOMI datasets. We note that h_i and a_i refer to the number of neurons and activation function in the hidden layer i, respectively, where $i = 1, 2, 3, ..., 8$.

**Dataset**	h_1, a_1	h_2, a_2	h_3, a_3	h_4, a_4	h_5, a_5	h_6, a_6	h_7, a_7	h_8	Optimizer	η	B	Tuner	α	γ
**TX Full**	60, RL	60, TH	41, TH	-	-	-	-	-	Adam	0.001	8192	BO	0.5	1.75
**TX AA**	60, RL	60, TH	60, TH	-	-	-	-	-	Adam	0.001	8192	HB	0.5	1.25
**TX NA**	60, TH	36, TH	41, RL	41, RL	36, TH	30, TH	-	-	SGD	0.0001	2048	RS	0.5	1.25
**OK Full**	60, TH	60, TH	-	-	-	-	-	-	Adam	0.001	8192	BO	0.5	1.25
**OK AA**	60, TH	60, RL	45, RL	30, TH	-	-	-	-	Adam	0.0001	1024	HB	0.5	1.25
**OK NA**	41, TH	41, RL	45, TH	41, RL	41, TH	-	-	-	NAdam	0.00001	1024	RS	0.5	1.25
**MOMI**	60, RL	30, TH	-	-	-	-	-	-	Adam	0.001	4096	BO	0.5	0.75
**MOMI AA**	30, RL	41, TH	45, TH	-	-	-	-	-	NAdam	0.001	512	BO	0.5	0.25

S14 Table. CSDNN-WCE (with Balanced Batches) architecture for Texas PUDF, Oklahoma PUDF and MOMI datasets. We note that h_i and a_i refer to the number of neurons and activation function in the hidden layer i, respectively, where $i = 1, 2, 3, ..., 8$.

**Dataset**	h_1, a_1	h_2, a_2	h_3, a_3	h_4, a_4	h_5, a_5	h_6, a_6	h_7, a_7	h_8, a_8	Optimizer	η	B	Tuner
**TX Full**	41, TH	60, RL	41, RL	30, RL	30, RL	-	-	-	Adam	0.001	8192	BO
**TX AA**	60, TH	30, RL	45, RL	-	-	-	-	-	RMSProp	0.001	8192	HB
**TX NA**	41, RL	30, RL	36, TH	45, TH	-	-	-	-	RMSProp	0.00001	2048	RS
**OK Full**	30, RL	30, TH	60, TH	45, RL	30, RL	60, RL	-	-	RMSProp	0.001	1024	BO
**OK AA**	30, TH	30, TH	45, RL	60, TH	-	-	-	-	Adam	0.0001	1024	HB
**OK NA**	60, TH	41, TH	45, RL	45, TH	41, TH	60, TH	36, RL	-	SGD	0.00001	1024	HB
**MOMI**	60, TH	60, TH	-	-	-	-	-	-	NAdam	0.001	4096	BO
**MOMI AA**	41, RL	30, TH	41, RL	36, TH	60, RL	-	-	-	RMSprop	0.001	819	BO

S15 Table. DNN (with Balanced Batches) architecture for Texas PUDF, Oklahoma PUDF and MOMI datasets. We note that h_i and a_i refer to the number of neurons and activation function in the hidden layer i, respectively, where $i = 1, 2, 3, ..., 8$.

**Dataset**	h_1, a_1	h_2, a_2	h_3, a_3	h_4, a_4	h_5, a_5	h_6, a_6	h_7, a_7	h_8	Optimizer	η	B	Tuner
**TX Full**	60, RL	36, RL	-	-	-	-	-	-	Adam	0.001	8192	HB
**TX AA**	60, TH	30, RL	45, RL	-	-	-	-	-	RMSProp	0.001	8192	HB
**TX NA**	60, TH	30, RL	45, RL	-	-	-	-	-	RMSProp	0.001	2048	HB
**OK Full**	60, TH	60, TH	60, TH	60, TH	-	-	-	-	Adam	0.001	1024	BO
**OK AA**	30, RL	41, TH	-	-	-	-	-	-	RMSProp	0.0001	1024	HB
**OK NA**	60, RL	36, TH	30, RL	41, RL	-	-	-	4-	NAdam	0.0001	1024	BO
**MOMI**	60, TH	60, TH	60, TH	-	-	-	-	-	Adam	0.001	4096	BO
**MOMI AA**	60, RL	60, RL	-	-	-	-	-	-	Adam	0.001	512	HB

We have investigated the relationship between neural network training convergence and the number of epochs. For neural network models, it is common to examine learning curve graphs to decide on model convergence. We specifically looked at the training and validation AUC versus the number of epoch plots in order to find the optimal number of epochs where the models start overfitting the data.

Figs S1 Fig-S6 Fig show the AUC of CSDNN-FL, CSDNN-WCE, and DNN models across a large number of epochs (up to 200) for both training and validation datasets. In most cases, the AUC increased quickly and eventually plateaued out. The training AUC and validation AUC tend to diverge with validation AUC stabilizing and sharp increase at the training AUC at a rapid pace for both the CSDNN-FL and CSDNN-WCE models at the optimal number of epochs. From these figures, it can be observed that the optimal number of epochs for the CSDNN-FL, CSDNN-WCE, and DNN in the Texas dataset is 30, 30, and 90 respectively. From the Texas validation and training AUC as shown in Figs. S1 Fig-S2 Fig, it can be observed that the validation AUC of both CSDNN-FL and CSDNN-WCE models leveled off very quickly and stabilized around the 30th epoch, while for the DNN model it did not stabilized until epoch 90, instead experienced fluctuations in AUC early in the training. From Figs. S3 Fig-S4 Fig, it can be observed that the optimal number of epochs for the CSDNN-FL, CSDNN-WCE, and DNN in the Oklahoma dataset is 20, 25, and 50 respectively. The validation AUC of the CSDNN-WCE increased quickly and stabilized at 25th epoch, while the CSDNN-FL stabilized at roughly the 50th epoch. The DNN model had the most noticable divergence in performance between training and validation AUC and stabilized at the 50th epoch. From Figs. S5 Fig-S6 Fig, it can be observed that the optimal number of epochs for the CSDNN-FL, CSDNN-WCE, and DNN in the MOMI dataset is 20, 20, and 60 respectively. The MOMI data resulted in the most similar trends between models, with both CSDNN-FL and CSDNN-WCE models reached their peak performance at about the 20th epoch, but with the CSDNN-FL reaching this level at a rapid pace, followed by CSDNN-WCE and DNN. Less variability in performance is seen in the MOMI dataset.

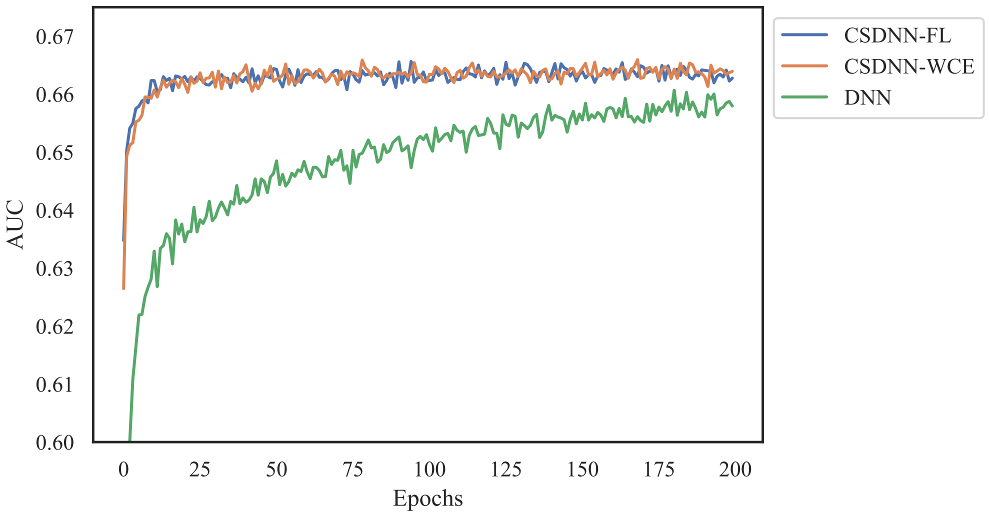

S1 Fig. Training AUC versus the number of epochs for the Texas dataset

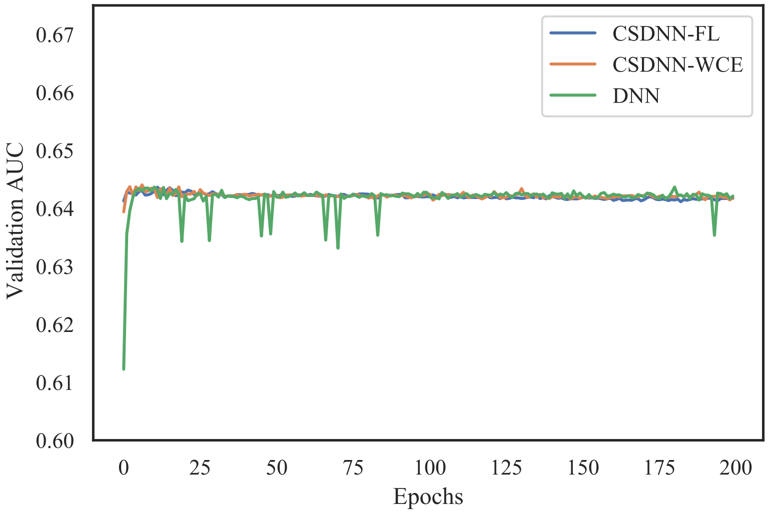

S2 Fig. Validation AUC versus the number of epochs for the Texas dataset

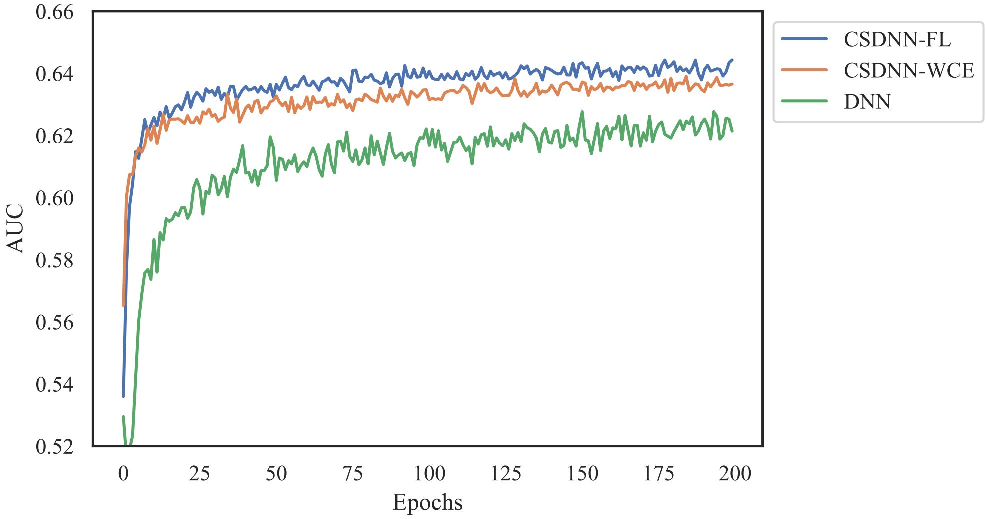

S3 Fig. Training AUC versus the number of epochs for the Oklahoma dataset

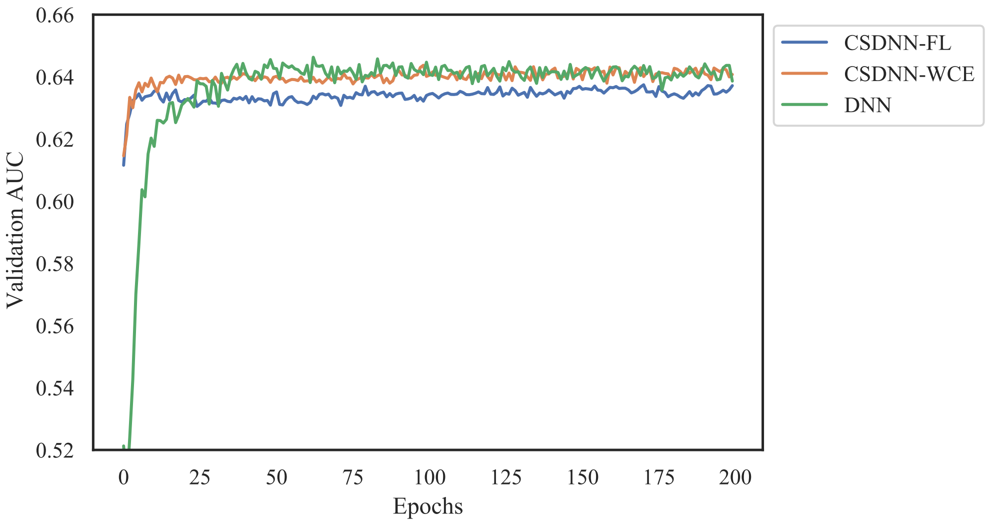

S4 Fig. Validation AUC versus the number of epochs for the Oklahoma dataset

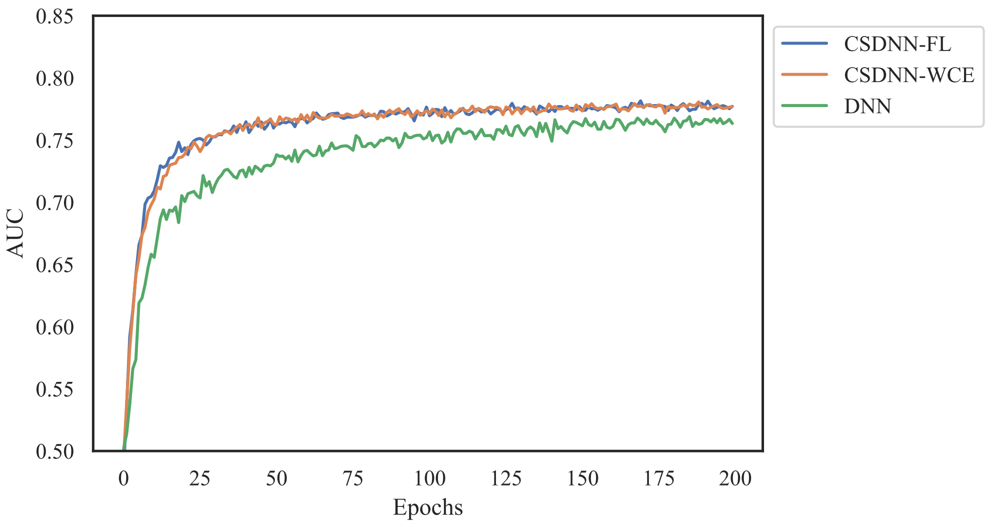

S5 Fig. Training AUC versus the number of epochs for the MOMI dataset

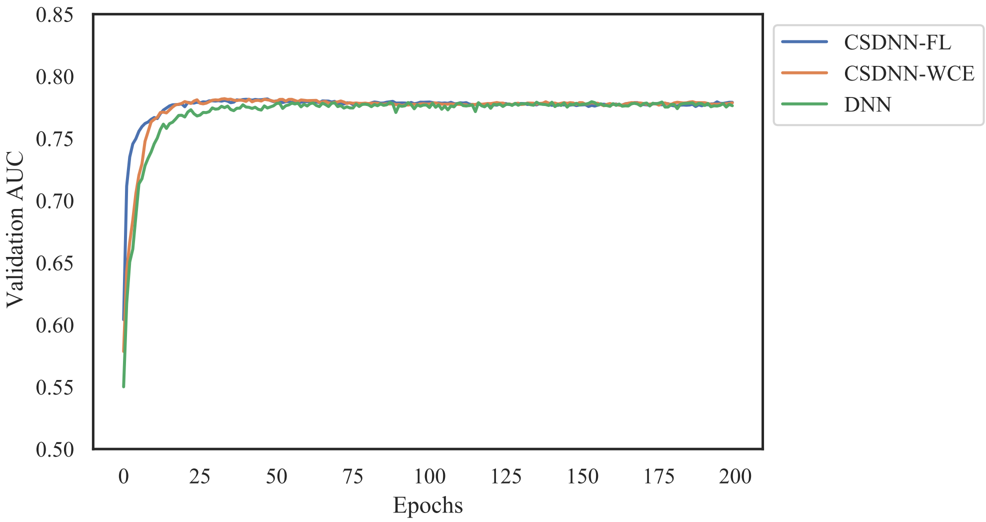

S6 Fig. Validation AUC versus the number of epochs for the MOMI dataset

S16 Table. Results of Wilcoxon rank sum test between CSDNN-FL and CSDNN-WCE, and benchmark methods for Texas data

**Comparison**	**p-value**	**Hypothesis** ($\alpha = 0.0005$)
**CSDNN-FL** > **CSDNN-WCE**	$\ll 0.0005$	Rejected H_0
**CSDNN-FL** > **DNN**	$\ll 0.0005$	Rejected H_0
**CSDNN-FL** > **LR**	$\ll 0.0005$	Rejected H_0
**CSDNN-FL** > **WLR**	$\ll 0.0005$	Rejected H_0
**CSDNN-FL** > **SVM-Lin**	$\ll 0.0005$	Rejected H_0
**CSDNN-FL** > **WSVM-Lin**	$\ll 0.0005$	Rejected H_0
**CSDNN-FL** > **SVM-RBF**	$\ll 0.0005$	Rejected H_0
**CSDNN-FL** > **WSVM-RBF**	$\ll 0.0005$	Rejected H_0
**CSDNN-FL** > **CSDNN-FL-BB**	$\ll 0.0005$	Rejected H_0
**CSDNN-FL** > **CSDNN-WCE-BB**	$\ll 0.0005$	Rejected H_0
**CSDNN-FL** > **DNN-BB**	$\ll 0.0005$	Rejected H_0
**CSDNN-WCE** > **DNN**	$\ll 0.0005$	Rejected H_0
**CSDNN-WCE** > **LR**	$\ll 0.0005$	Rejected H_0
**CSDNN-WCE** > **WLR**	$\ll 0.0005$	Rejected H_0
**CSDNN-WCE** > **SVM-Lin**	$\ll 0.0005$	Rejected H_0
**CSDNN-WCE** > **WSVM-Lin**	$\ll 0.0005$	Rejected H_0
**CSDNN-WCE** > **SVM-RBF**	$\ll 0.0005$	Rejected H_0
**CSDNN-WCE** > **WSVM-RBF**	0.999	Not rejected H_0
**CSDNN-WCE** > **CSDNN-FL-BB**	$\ll 0.0005$	Rejected H_0
**CSDNN-WCE** > **CSDNN-WCE-BB**	$\ll 0.0005$	Rejected H_0
**CSDNN-WCE** > **DNN-BB**	$\ll 0.0005$	Rejected H_0

S17 Table. Results of Wilcoxon rank sum test between CSDNN-FL and CSDNN-WCE, and benchmark methods for Oklahoma data.

Comparison	p-value	Hypothesis ($\alpha = 0.0005$)
**CSDNN-FL** $>$ **CSDNN-WCE**	$\ll 0.0005$	Rejected H_0
**CSDNN-FL** $>$ **DNN**	$\ll 0.0005$	Rejected H_0
**CSDNN-FL** $>$ **LR**	$\ll 0.0005$	Rejected H_0
**CSDNN-FL** $>$ **WLR**	$\ll 0.0005$	Rejected H_0
**CSDNN-FL** $>$ **SVM-Lin**	$\ll 0.0005$	Rejected H_0
**CSDNN-FL** $>$ **WSVM-Lin**	$\ll 0.0005$	Rejected H_0
**CSDNN-FL** $>$ **SVM-RBF**	$\ll 0.0005$	Rejected H_0
**CSDNN-FL** $>$ **WSVM-RBF**	$\ll 0.0005$	Rejected H_0
**CSDNN-FL** $>$ **CSDNN-FL-BB**	$\ll 0.0005$	Rejected H_0
**CSDNN-FL** $>$ **CSDNN-WCE-BB**	$\ll 0.0005$	Rejected H_0
**CSDNN-FL** $>$ **DNN-BB**	$\ll 0.0005$	Rejected H_0
**CSDNN-WCE** $>$ **DNN**	$\ll 0.0005$	Rejected H_0
**CSDNN-WCE** $>$ **LR**	$\ll 0.0005$	Rejected H_0
**CSDNN-WCE** $>$ **WLR**	0.630	Not rejected H_0
**CSDNN-WCE** $>$ **SVM-Lin**	$\ll 0.0005$	Rejected H_0
**CSDNN-WCE** $>$ **WSVM-Lin**	$\ll 0.0005$	Rejected H_0
**CSDNN-WCE** $>$ **SVM-RBF**	$\ll 0.0005$	Rejected H_0
**CSDNN-WCE** $>$ **WSVM-RBF**	$\ll 0.0005$	Rejected H_0
**CSDNN-WCE** $>$ **CSDNN-FL-BB**	0.999	Not rejected H_0
**CSDNN-WCE** $>$ **CSDNN-WCE-BB**	$\ll 0.0005$	Rejected H_0
**CSDNN-WCE** $>$ **DNN-BB**	0.989	Not rejected H_0

S18 Table. Results of Wilcoxon rank sum test between CSDNN-FL and CSDNN-WCE, and benchmark methods for MOMI data.

Comparison	p-value	Hypothesis ($\alpha = 0.0005$)
**CSDNN-FL** $>$ **CSDNN-WCE**	0.99	Not rejected H_0
**CSDNN-FL** $>$ **DNN**	$\ll 0.0005$	Rejected H_0
**CSDNN-FL** $>$ **LR**	$\ll 0.0005$	Rejected H_0
**CSDNN-FL** $>$ **WLR**	0.999	Not rejected H_0
**CSDNN-FL** $>$ **SVM-Lin**	$\ll 0.0005$	Rejected H_0
**CSDNN-FL** $>$ **WSVM-Lin**	0.004	Not rejected H_0
**CSDNN-FL** $>$ **SVM-RBF**	$\ll 0.0005$	Rejected H_0
**CSDNN-FL** $>$ **WSVM-RBF**	0.960	Not rejected H_0
**CSDNN-FL** $>$ **CSDNN-FL-BB**	0.956	Not rejected H_0
**CSDNN-FL** $>$ **CSDNN-WCE-BB**	$\ll 0.0005$	Rejected H_0
**CSDNN-FL** $>$ **DNN-BB**	0.999	Not rejected H_0
**CSDNN-WCE** $>$ **DNN**	$\ll 0.0005$	Rejected H_0
**CSDNN-WCE** $>$ **LR**	$\ll 0.0005$	Rejected H_0
**CSDNN-WCE** $>$ **WLR**	0.999	Not rejected H_0
**CSDNN-WCE** $>$ **SVM-Lin**	$\ll 0.0005$	Rejected H_0
**CSDNN-WCE** $>$ **WSVM-Lin**	0.001	Not rejected H_0
**CSDNN-WCE** $>$ **SVM-RBF**	$\ll 0.0005$	Rejected H_0
**CSDNN-WCE** $>$ **WSVM-RBF**	0.995	Not rejected H_0
**CSDNN-WCE** $>$ **CSDNN-FL-BB**	0.003	Rejected H_0
**CSDNN-WCE** $>$ **CSDNN-WCE-BB**	$\ll 0.0005$	Rejected H_0
**CSDNN-WCE** $>$ **DNN-BB**	0.616	Not rejected H_0

S19 Table. Results of Wilcoxon rank sum test between CSDNN-FL and CSDNN-WCE, and benchmark methods for Texas African American data.

**Comparison**	**p-value**	**Hypothesis** ($\alpha = 0.0005$)
**CSDNN-FL** $>$ **CSDNN-WCE**	$\ll 0.0005$	Rejected H_0
**CSDNN-FL** $>$ **DNN**	$\ll 0.0005$	Rejected H_0
**CSDNN-FL** $>$ **FL-BB**	$\ll 0.0005$	Rejected H_0
**CSDNN-FL** $>$ **WCE-BB**	$\ll 0.0005$	Rejected H_0
**CSDNN-FL** $>$ **CE-BB**	$\ll 0.0005$	Rejected H_0
**CSDNN-WCE** $>$ **DNN**	$\ll 0.0005$	Rejected H_0
**CSDNN-WCE** $>$ **CSDNN-FL-BB**	0.999	Not rejected H_0
**CSDNN-WCE** $>$ **CSDNN-WCE-BB**	$\ll 0.0005$	Rejected H_0
**CSDNN-WCE** $>$ **DNN-BB**	0.999	Not rejected H_0

S20 Table. Results of Wilcoxon rank sum test between CSDNN-FL and CSDNN-WCE, and benchmark methods for Oklahoma African American data.

**Comparison**	**p-value**	**Hypothesis** ($\alpha = 0.0005$)
**CSDNN-FL** $>$ **CSDNN-WCE**	0.862	Not rejected H_0
**CSDNN-FL** $>$ **DNN**	$\ll 0.0005$	Rejected H_0
**CSDNN-FL** $>$ **CSDNN-FL-BB**	$\ll 0.0005$	Rejected H_0
**CSDNN-FL** $>$ **CSDNN-WCE-BB**	$\ll 0.0005$	Rejected H_0
**CSDNN-FL** $>$ **DNN-BB**	$\ll 0.0005$	Rejected H_0
**CSDNN-WCE** $>$ **DNN**	$\ll 0.0005$	Rejected H_0
**CSDNN-WCE** $>$ **CSDNN-FL-BB**	$\ll 0.0005$	Rejected H_0
**CSDNN-WCE** $>$ **CSDNN-WCE-BB**	$\ll 0.0005$	Rejected H_0
**CSDNN-WCE** $>$ **DNN-BB**	$\ll 0.0005$	Rejected H_0

S21 Table. Results of Wilcoxon rank sum test between CSDNN-FL and CSDNN-WCE, and benchmark methods for Texas Native American data.

**Comparison**	**p-value**	**Hypothesis** ($\alpha = 0.0005$)
**CSDNN-Focal** $>$ **CSDNN-WCE**	$\ll 0.0005$	Rejected H_0
**CSDNN-Focal** $>$ **DNN**	$\ll 0.0005$	Rejected H_0
**CSDNN-Focal** $>$ **FL-BB**	$\ll 0.0005$	Rejected H_0
**CSDNN-Focal** $>$ **WCE-BB**	$\ll 0.0005$	Rejected H_0
**CSDNN-Focal** $>$ **CE-BB**	$\ll 0.0005$	Rejected H_0
**CSDNN-WCE** $>$ **DNN**	$\ll 0.0005$	Rejected H_0
**CSDNN-WCE** $>$ **FL-BB**	$\ll 0.0005$	Rejected H_0
**CSDNN-WCE** $>$ **WCE-BB**	$\ll 0.0005$	Rejected H_0
**CSDNN-WCE** $>$ **CE-BB**	0.141	Not rejected H_0

S22 Table. Results of Wilcoxon rank sum test between CSDNN-FL and CSDNN-WCE, and benchmark methods for Oklahoma Native American data.

**Comparison**	**p-value**	**Hypothesis** ($\alpha = 0.0005$)
**CSDNN-FL** $>$ **CSDNN-WCE**	0.007	Not rejected H_0
**CSDNN-FL** $>$ **DNN**	$\ll 0.0005$	Rejected H_0
**CSDNN-FL** $>$ **CSDNN-FL-BB**	$\ll 0.0005$	Rejected H_0
**CSDNN-FL** $>$ **CSDNN-WCE-BB**	$\ll 0.0005$	Rejected H_0
**CSDNN-FL** $>$ **DNN-BB**	$\ll 0.0005$	Rejected H_0
**CSDNN-WCE** $>$ **DNN**	$\ll 0.0005$	Rejected H_0
**CSDNN-WCE** $>$ **CSDNN-FL-BB**	$\ll 0.0005$	Rejected H_0
**CSDNN-WCE** $>$ **CSDNN-WCE-BB**	$\ll 0.0005$	Rejected H_0
**CSDNN-WCE** $>$ **DNN-BB**	0.0004	Rejected H_0

S23 Table. Results of Wilcoxon rank sum test between CSDNN-FL and CSDNN-WCE, and benchmark methods for MOMI African American data.

Comparison	p-value	Hypothesis ($\alpha = 0.0005$)
**CSDNN-FL** > **CSDNN-WCE**	0.998	Not rejected H_0
**CSDNN-FL** > **DNN**	$\ll 0.0005$	Rejected H_0
**CSDNN-FL** > **CSDNN-FL-BB**	0.999	Not rejected H_0
**CSDNN-FL** > **CSDNN-WCE-BB**	0.003	Rejected H_0
**CSDNN-FL** > **DNN-BB**	0.207	Not rejected H_0
**CSDNN-WCE** > **DNN**	$\ll 0.0005$	Rejected H_0
**CSDNN-WCE** > **CSDNN-FL-BB**	0.755	Not rejected H_0
**CSDNN-WCE** > **CSDNN-WCE-BB**	$\ll 0.0005$	Rejected H_0
**CSDNN-WCE** > **DNN-BB**	$\ll 0.0005$	Rejected H_0
